# Weighted average ensemble-based semantic segmentation in biological electron microscopy images

**DOI:** 10.1007/s00418-022-02148-3

**Published:** 2022-08-20

**Authors:** Kavitha Shaga Devan, Hans A. Kestler, Clarissa Read, Paul Walther

**Affiliations:** 1 Central Facility of Electron Microscopy, Ulm University, Albert Einstein-Allee 11, 89081 Ulm, Germany; 2 Medical Systems Biology, Ulm University, Albert Einstein-Alee 11, 89081 Ulm, Germany; 3 Institute of Virology, Ulm University Medical Center, Albert Einstein-Allee 11, 89081 Ulm, Germany

**Keywords:** Artificial intelligence, Deep learning, Automated image analysis, Electron microscopy, Semantic segmentation, Ensemble-based machine learning

## Abstract

Semantic segmentation of electron microscopy images using deep learning methods is a valuable tool for the detailed analysis of organelles and cell structures. However, these methods require a large amount of labeled ground truth data that is often unavailable. To address this limitation, we present a weighted average ensemble model that can automatically segment biological structures in electron microscopy images when trained with only a small dataset. Thus, we exploit the fact that a combination of diverse base-learners is able to outperform one single segmentation model. Our experiments with seven different biological electron microscopy datasets demonstrate quantitative and qualitative improvements. We show that the Grad-CAM method can be used to interpret and verify the prediction of our model. Compared with a standard U-Net, the performance of our method is superior for all tested datasets. Furthermore, our model leverages a limited number of labeled training data to segment the electron microscopy images and therefore has a high potential for automated biological applications.

**Supplementary Information:**

The online version contains supplementary material available at 10.1007/s00418-022-02148-3.

## Introduction

Comprehensive analysis of organelles, cell structures, and viral particles leads to understanding of various pathological processes, enabling discoveries, and insights into disease mechanisms. Electron microscopy (EM) has been proven to be a valuable method for biologists to analyze these biological structures in high resolution. In recent years, advancements in this field have enabled the acquisition of large volumes of three-dimensional EM data (Villinger et al. 2014; Kubota et al. 2018; Zheng et al. 2018; Maniates-Selvin et al. 2020; Read et al. 2021). To quantify these large volumes of EM data, biologists have routinely used segmentation tools to obtain critical information about the morphological parameters of the organelles, cell structures, and viral particles. However, manual segmentation of large EM datasets requires expert input, and is tedious as well as labor-intensive. To address this issue, attempts have been made to develop semi-automated segmentation methods using machine learning approaches such as ilastik (Sommer et al. 2011) and DeepMIB (Belevich et al. 2016).

While these approaches successfully improved the rate of segmentation, a considerable amount of manual interaction is required for corrections and quality control. This is because the inherently low contrast in EM images and the variations in appearances in biological structures and sample preparation artifacts could lead to segmentation inaccuracies. Therefore, the lack of fully automatic methods for EM segmentation impedes the routine use for quantitative analysis of biological structures, and there is a demand for the development of accurate and efficient tools for the automatic quantification of EM images.

Recently, deep learning (DL) methods including the above-mentioned approaches have shown incredible success in a wide variety of biomedical image analysis applications (Webb 2018; Ching et al. 2018; Tang et al. 2019; Tian et al. 2021). Convolution neural networks (CNNs) are a class of deep neural networks, which are most commonly applied to analyze visual images. The main advantage of the CNN approach is that it utilizes raw images and expert-labeled data instead of hand-crafted feature vectors that require a high level of domain expertise. As a result, it can automatically learn high-level discriminant features for visual pattern recognition tasks from the input data (Tajbakhsh et al. 2016). The success of CNNs has prompted the development of several deep architectures for the semantic segmentation of images, whereby each pixel is classified into a specific class. CNN-based semantic segmentation has been proven to outperform traditional segmentation methods (Mahony et al. 2020).

In this context, U-Net (Ronneberger et al. 2015) has been widely used by the biomedical image analysis community and is regarded as one of the most successful architectures for semantic segmentation. Heinrich et al. (2021) developed a U-Net-based 3D segmentation model that is able to segment 35 different cellular organelle classes in focused ion beam scanning electron microscopy (FIB-SEM) images. Modified versions of U-Net have also been successfully used to segment various biological structures such as cells, small extracellular vesicles, and mitochondria in EM images (Casser et al. 2020; Fischer et al. 2020).

Monchot et al. (2021) utilized the Mask R-CNN architecture to segment titanium dioxide particles in the form of agglomerates in scanning electron microscopy (SEM) images. SegNet, a Bayesian-based architecture, was introduced by Koobragade and Agarwal (2018) to carry out multi-class segmentation in serial section transmission electron microscopy (ssTEM) images. George et al. (2021) developed CASSPER, a DL tool for the automated segmentation of protein particles in cryogenic transmission electron microscopy (cryo-TEM) images. The rise in the application of deep CNNs for EM images prompted Kharabag (2021) to compare the performance of four DL architectures for the semantic segmentation of HeLa cells in serial block-face scanning electron microscopy (SBFSEM) images. On the other hand, Horwath et al. (2020) studied a variety of CNN architectures to define the most important features of DL models for the efficient segmentation of structures in TEM images.

Most of the currently available semantic segmentation methods require a large number of representative ground truth data, in order to be successful and generalize well on unseen images. Generalization is the ability of the model to perform well on unseen data and is an essential characteristic of a successful DL model. Therefore, a large number of images have to be manually labeled to provide sufficient ground truth for algorithm learning. However, labeling large amounts of data in the biological field requires expert knowledge, is extremely time-consuming, and also expensive. Moreover, the task is prone to inter- and intra-user bias. It therefore could lead to poor quality of ground truth images, especially when many images need to be manually labeled. The low availability of large labeled training data might be the reason that automatic analysis of EM images based on deep learning approaches are not yet routinely applied in the field of biomedical EM.

To mitigate this issue, researchers have been studying various techniques for segmenting EM images with a small labeled dataset and still obtaining performance that is on par with human experts. Roels and Saeys (2019) proposed a method for cost-efficient segmentation of electron microscopy images using active learning by smartly selecting the samples that require labeling. Generative adversarial networks were used by Shaga Devan et al. (2021) for the generation of synthetic EM images as a data augmentation method which improved the detection and localization of herpesvirus human cytomegalovirus (HCMV). The authors of EM-Net developed a scalable deep neural network ensemble for rapid learning from ground-truth data for binary image segmentation (Khadangi et al. 2021). The model was trained and tested with two binary datasets. For the ensemble process, multiple models were created with various batch normalizations modifications and combined for final prediction.

Our work was inspired by CDeep3M (Haberl et al. 2018), which is a ready-to-use large-scale image segmentation tool employing cloud-based deep convolutional neural networks for the segmentations of biological structures from both electron and light microscopy modalities. This tool was developed using an ensemble structure integrating models trained with one, three, and five consecutive image frames and utilizes the concept of transfer learning. For performing segmentations, users are provided with a series of pre-trained networks for structures such as mitochondria, synapses, membranes, and vesicles that were originally trained on electron tomography and serial block-face scanning electron microscopy (SBEM) images.

While the availability of a plug-and-play segmentation tool such as CDeep3M is indeed revolutionary and extremely helpful to the biological EM community, it may not be able to segment a large variety of biological structures due to the intra-class variations and inter-class similarities in appearances that is inherent in many viral particles, cells and organelles. EM images appearances also often vary due to image acquisition, noise, and sample preparations artifacts that could lead to segmentation inaccuracies which will then still require pre- and post-processing user intervention.

Therefore, encouraged by this tool, we aim to develop a semantic segmentation model for the multi-class segmentation of biological structures in EM images using user customized limited labeled ground truth training datasets curated by biologists. In this work, we explore the segmentation of biological structures such as cytoplasm, nucleus, mitochondria, and chromosomes in both TEM and SEM images. Our goal is to provide the biologists with a segmentation model that can be easily adapted to their own EM image datasets as well as biological structures of choice. These enables biologists to have flexibility and control in the usage of deep learning for their customized segmentation needs without the need for large unaffordable labeling efforts. To this end, we have explored the concept of DL ensembles for improving segmentation quality. We investigated the feasibility of using off-the-shelf ImageNet (Russakovsky et al. 2015) pre-trained networks for our ensemble model which differed from the method used by Haberl (2018).

While there is no standard size requirement for DL training datasets, generally, the larger the training dataset, the better is the ability of the CNN to learn. Therefore, CNNs do not perform well when trained with a small amount of training data (Tajbakhsh et al. 2016). To overcome this limitation, our proposed method utilizes a CNN-based weighted averaging ensemble approach that can learn complex discriminant features from a small dataset to segment biological structures in both TEM and SEM images. The amount of training images in all of the datasets used in this work range from 66 to 258. The primary motivation for using a weighted average ensemble approach is that it has better predictive performance compared than a single model since it produces a lower error rate and reduces variance (Mustafa et al. 2020). However, a single model will not capture the entire underlying structure of the data to achieve optimal predictions. Therefore, by combining multiple base-learners, more information can be captured of the data’s underlying structure and can significantly improve prediction accuracy (Shahhosseini et al. 2021).

We refer to our proposed model as WAE-Net (weighted average ensemble network). It is built from an ensemble of U-Nets, each of which is trained with a different pre-trained network and, their predictions are combined in a weighted average manner for the multi-class segmentation of EM images. To provide a deeper understanding of the segmentation results given by the ensemble model, we further applied a visual CNN interpretation approach called Gradient-weighted Class Activation Mapping (Grad-CAM) (Selvaraju et al. 2017) to identify critical regions in the images for prediction. We compared our proposed approach against the standard U-Net for quantitative and qualitative performance. Our ensemble method enabled us to obtain better segmentation results than the U-Net. Taken together, we have developed an approach to perform end-to-end multi-class segmentation of EM images using small datasets allowing us to leverage the benefits of current DL developments without needing unaffordable extensive labeling efforts.

## Materials and methods

### Image dataset

We evaluated our proposed method using seven biological EM image datasets encompassing two types of EM imaging modalities; TEM and SEM for both training and testing. Datasets 1–3 are biological cells prepared by high-pressure freezing, freeze substitution, and plastic embedding. The EM datasets were obtained with two different approaches. The first approach is to mount the embedded cells in a scanning EM additionally equipped with a focused ion beam (FIB-SEM). A small portion of the embedded cell is removed with the focused ion beam and the newly produced surface is imaged with the scanning EM. This process is repeated several hundred times and the images can then be reconstructed to a three-dimensional model (Villinger et al. 2012). We have used this approach to obtain dataset 1. The second approach is to section the plastic embedded cell using an ultra-microtome equipped with a diamond knife and then taking images of each section with a TEM, which was used to obtain datasets 2 and 3 (Villinger et al. 2014). Datasets 1–3 are comprised of two-dimensional images obtained from three-dimensional samples; a biological cell embedded in a plastic that is then sectioned either with a diamond knife or an ion beam. Datasets 4–7 were publicly available two-dimensional image datasets from Morath et al. (2013). All the images used in this work, were processed as two-dimensional images. The details of each dataset are listed in Table 1.

**Table 1:** Summary of details about the datasets used in this work

Dataset	Specimen	Imaging modality	Pixel resolution (nm)	Structures for segmentation
Dataset 1	Human pancreatic carcinoid cell line	FIB-SEM	9	Cytoplasm, nucleus
Dataset 2	BON cell during interphase	Serial section TEM	9	Cytoplasm, chromosomes
Dataset 3	BON cell during mitosis	Serial section TEM	26.3	Cytoplasm, nucleus, mitochondria
Dataset 4	Human T-cell line Jurkat	TEM	6.41	Cytoplasm, nucleus
Dataset 5	Primary human T-cell blood	TEM	2.33	Cytoplasm, nucleus
Dataset 6	Murine B-cell line J558L	TEM	15.26	Cytoplasm, nucleus
Dataset 7	Phytohemagglutinin/IL-2 expanded human T cells	TEM	15.74	Cytoplasm, nucleus

These seven datasets contain diverse images, which differ in biological structures, imaging modality, pixel resolution, contrast, brightness, and noise levels to account for the variability typical for biological EM. All seven datasets, containing whole-slice images, were pixel-wise manually labeled by biologists and serve as the ground truth for network training and testing. These datasets were randomly divided into a training and hold-out test set with a ratio of 8:2. In order to avoid bias, a 5 × 5-fold cross validation was performed during model training. The hold-out test set was only used for testing. Table 2 shows the details of the distribution of images for training and testing and the number of segmentation classes for all the datasets. In this work, the background, which comprises all structures that are not of interest in the EM images, is also considered a segmentation class.

**Table 2:** Number of segmentation classes, total images, training images and test images for all datasets

Dataset	Classes for segmentation	Total images	Training images	Test images
Dataset 1	3	323	258	65
Dataset 2	3	167	133	34
Dataset 3	4	117	66	17
Dataset 4	3	135	108	27
Dataset 5	3	122	97	25
Dataset 6	3	115	92	23
Dataset 7	3	108	86	22

### Experimental methods pipeline

**Fig. 1:**
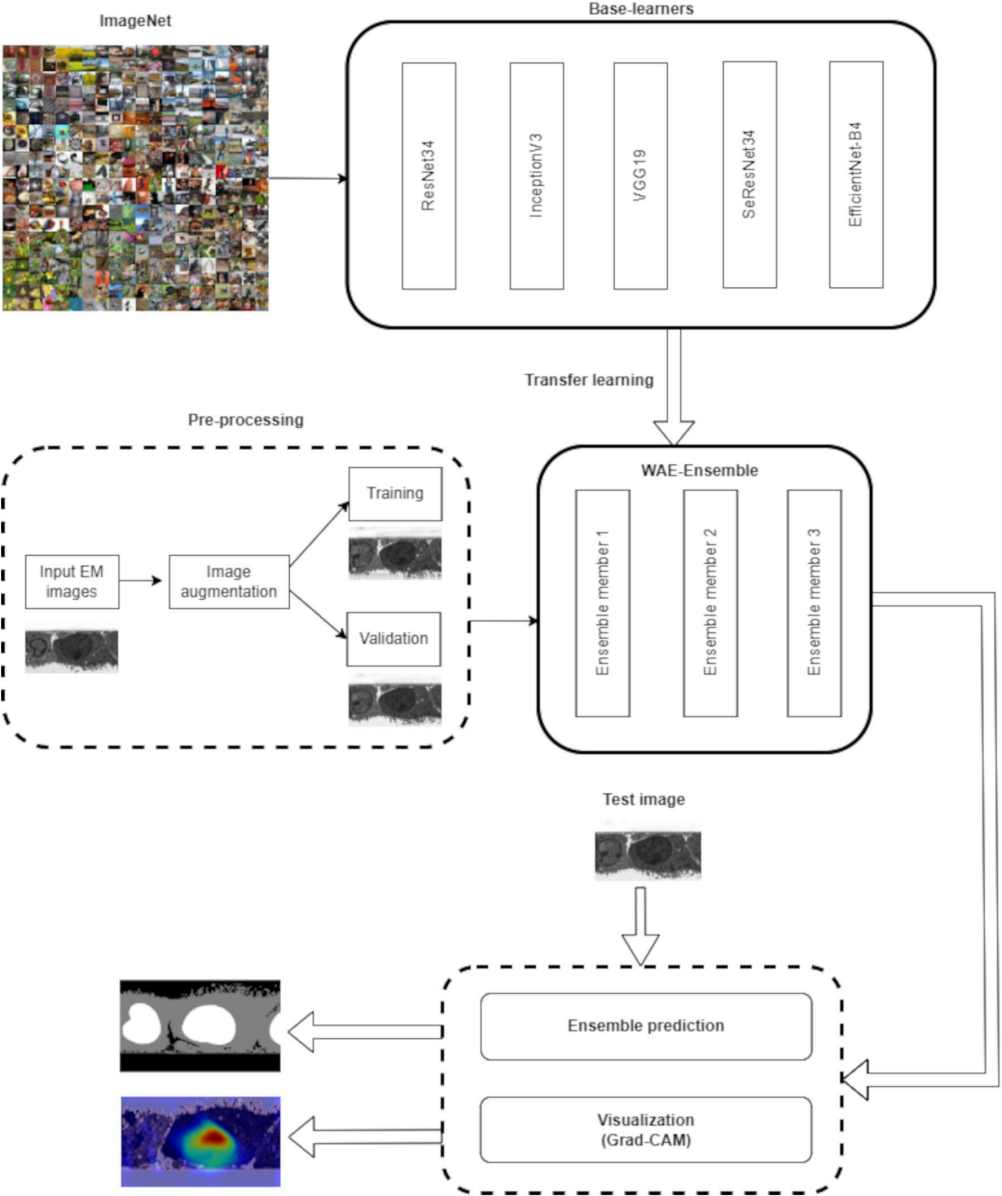
Schematic architecture of the proposed ensemble-based semantic segmentation of biological electron microscopy images. The individual base-learners were trained with the ImageNet dataset. Then, the top-three best-performing learners were combined in an ensemble. The ensemble model was further trained with the electron microscopy datasets and tested. The Grad-CAM was used to further verify the correctness of the predictions

A representative diagram illustrating the pipeline of our proposed weighted average ensemble model (WAE-Net) for the semantic segmentation of biological structures in EM images is shown in Fig. 1. All the tasks in the proposed model pipeline were done using a Tesla T4 GPU with Tensorflow 2.2.0, Keras 2.3.1 and Python 3.7.11.

### Base-learner construction and training

**Fig. 2:**
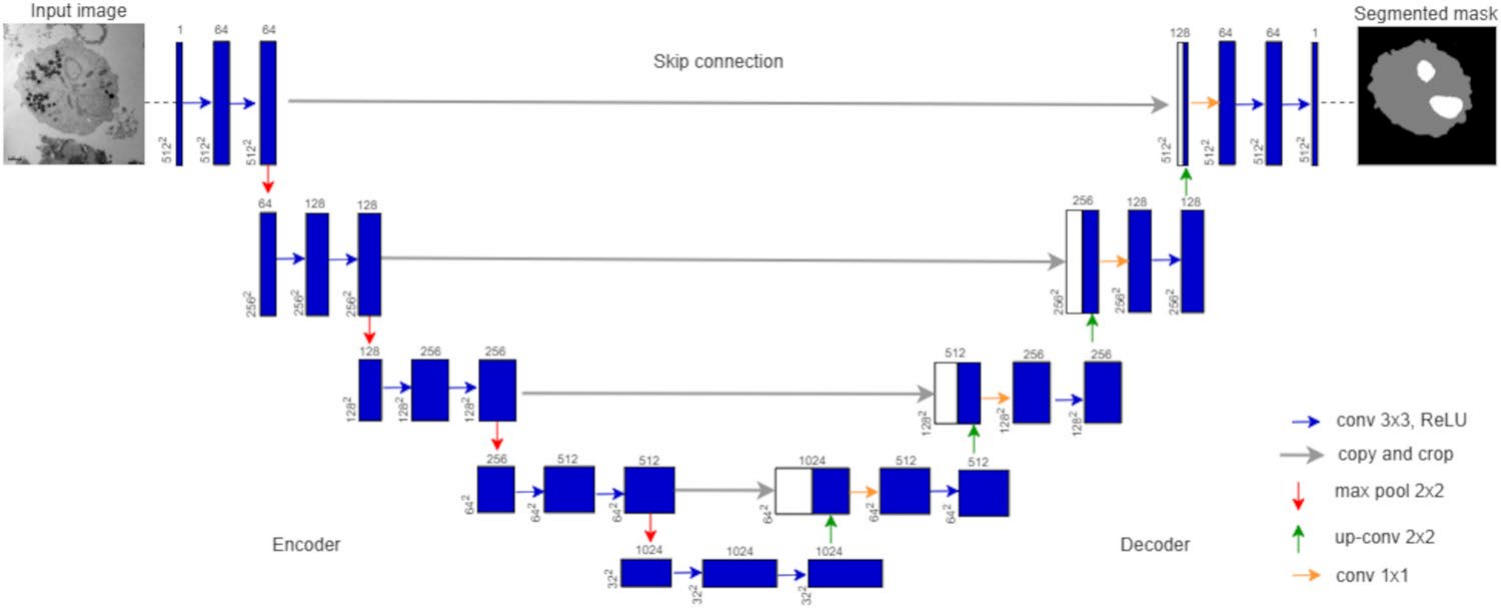
A standard U-Net architecture. The network consists of an encoder and decoder path. Each blue box corresponds to a multi-channel feature map. The number of channels for each feature map is denoted at the top of the box. The image dimension is provided at the lower-left edge of the box. White boxes represent copied feature maps. The arrows denote the various convolutional neural-network operations (Ronneberger et al. 2015)

We first trained five individual base-learners as ensemble members to construct the weighted average ensemble. The base-learners were created using the U-Net architecture. Figure 2 shows the typical architecture of a U-Net. Its architecture contains an encoder network that is followed by a decoder network (Ronneberger et al. 2015). The bottom-most layer at the base of the U-shaped network is the bottleneck section. The encoder is a classification network consisting of alternating convolution layers with rectified linear unit (ReLU) and batch normalizations, followed by maximum pooling-based downsampling operations which increases the number of feature maps per layer. The input images are encoded into feature representations at various levels.

The bottleneck layer provides mediation between the encoder and decoder layer. The decoder semantically projects the discriminative features learned by the encoder onto the pixel space to obtain a dense classification. It consists of upsampling of the feature maps followed by convolution operations. Upsampling is performed to restore the condensed feature maps to the original size of the input image. This is achieved by expanding the feature dimensions.

In the base-learners of our WAE-Net, short and long skip connections were used within the pre-trained encoders as well as between the encoders and decoders. Detailed diagrams indicating the convolutional layers and skip connections of our proposed model are given as Electronic Supplementary Material. For ResNet34, SeResNet34, and EfficientNetB4, short skip connections were used within the encoders to pass information from the initial layers to deeper layers via matrix additions. Specifically, activations and convolutional layers were short skip connected to the layers performing arithmetic operations (Electronic Supplementary Material). These skip connections provide an uninterrupted gradient flow from the first layer to the last layer, thereby mitigating the effects of the vanishing gradient problem, which is a common occurrence in deep architectures such as the encoders mentioned above. InceptionV3 and VGG19 architectures did not use short skip connections within their encoders.

Long skip connections were utilized in ResNet34, InceptionV3, VGG19, SeResNet34, and EfficientNetB4 pre-trained base-learners (Fig. 2). They were used to concatenate the feature maps from the encoder’s layer to the same scale feature maps of the decoder. Specifically, the activation layers in the encoder were connected to the corresponding concatenate layers in the decoder (Electronic Supplementary Material). This was done in order to compensate for the loss of spatial information in the encoder during the downsampling process. Taken together, the use of skip training in our WAE-Net helped to stabilize training and convergence. Simply stated, the encoder part encodes the semantics and contextual information of the input images while the decoder part uses this encoded information for the generation of segmentation maps.

To construct the individual base-learners, we replaced the encoder part of the U-Net with a pre-trained CNN network. The decoder part was maintained as depicted in Fig. 2. We built the encoder by removing the fully connected layers of the pre-trained networks and replacing them with a single convolutional layer of 1024 feature channels that serve as the bottleneck part of the base-learner, separating the encoder from the decoder. The output of the transposed convolution layers is then concatenated with the output of the corresponding part of the decoder. The resultant feature maps are treated by convolution operations to keep the number of channels the same to preserve symmetry of the network.

Pre-trained networks were used as encoders in order to leverage transfer learning for our segmentation goals. Devan et al. (2019) have shown that transfer learning is a highly effective performance booster for EM images when working with a small labeled dataset. It is the process of taking a pre-trained neural network and adapting the neural network to a different dataset by transferring its learned features. Therefore, in this work, the encoder was initialized with pre-trained weights from ImageNet database (Russakovsky et al. 2015) and trained on our own datasets.

The rationale for using pre-trained networks is that the imported network will already have sufficient knowledge in the broader aspects of images such as edge, texture, and shape information that could also be useful for EM image segmentation (Kolesnikov et al. 2020; Dhillon and Haque 2020). While the U-Net can also be trained from scratch on the ImageNet database, however, training the huge dataset, which contains over 14 million images, will require extremely high computational resources, which is a major limitation in many electron microscopy laboratories around the globe. Therefore, using pre-trained networks will be an optimized cost-effective solution. We selected five state-of-the-art pre-trained networks, and for each dataset, we replaced the encoder of the U-Net with each of the five networks, yielding five base-learners.

For an ensemble to outperform any of its members, the base-learners must be accurate and diverse enough to capture the structure of the data effectively. The diversity in the base learners is where the strength of the ensemble lies (Zhou 2009). Furthermore, different pre-trained networks have different properties and learning schemes that contribute towards the performance of the base-learners in the ensemble. Therefore, proper selection of pre-trained networks is imperative to the success of our ensemble model.

While many pre-trained networks are available, we selected these five based on three requirements. First, is that the networks have consistently given state-of-the-art performance. Secondly, the networks must have different architectural designs, and finally, the networks should only have a small number of parameters. While the first requirement is self-explanatory, the second requirement is crucial for creating diversity in our ensemble as different base learners have different properties and learning paradigms. A diverse ensemble can maximize the learning ability of the model while minimizing variance and bias. The final requirement regarding the network's parameters warrants deeper analysis.

**Fig. 3:**
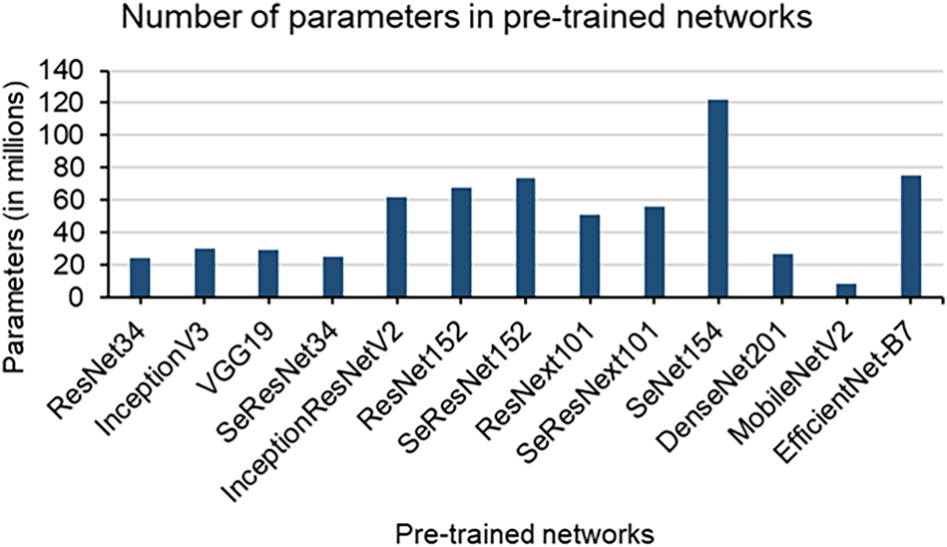
Graph depicting the total number of learning parameters in publicly available state-of-the-art pre-trained convolutional neural networks. The total number of parameters encompass both trainable and non-trainable learning parameters

Current CNN-based state-of-the-art networks have billions of learning parameters that provide highly competitive results when trained with large datasets. On the other hand, these large networks perform poorly when trained with small datasets. It has been observed that training large complex networks with small datasets often leads to overfitting (Ying 2019). Overfitting happens when noise or random fluctuations in the training data is picked up and learned as features by the network to the extent that it negatively impacts its performance and subsequently affects segmentation quality. Figure 3 shows a selection of commonly used state-of-the-art pre-trained networks and their corresponding number of parameters.

Based on Fig. 3, we have selected networks that yield good performance, are diverse in their learning approach, and have a total number of parameters of less than 30 million. A threshold of 30 million parameters was chosen because based on preliminary testing (data not shown), a network with a larger number of parameters overfit the datasets while a network with lower number of parameters did not learn effectively from the training data. Therefore, the pre-trained networks used as encoders in this work are ResNet34 (He et al. 2016), InceptionV3 (Szegedy et al. 2015), VGG19 (Simonyan and Zisserman 2015), SeResNet34 (Hu et al. 2019), and EfficientNet-B4 (Tan and Le 2020). Table 3 shows the details of the selected pre-trained networks. For each dataset, the above-mentioned five pre-trained networks were individually used as encoders in the U-Net and trained. This results in five individual base-learners for each dataset.

**Table 3:** Number of parameters in the selected pre-trained networks

Model	Trainable parameters	Non-trainable parameters	Total parameters
ResNet34	24,439,094	17,350	24,456,444
InceptionV3	36,416	29,896,979	29,933,395
VGG19	4032	29,058,227	26,062,259
SeResNet34	17,350	24,600,290	24,617,640
EfficientNet-B4	25,735,307	25,608,123	25,735,307

### Hyperparameter optimization

Hyperparameters are all the parameters that are set in a CNN model before starting the training process in order to configure the model to our dataset. Hyperparameter optimization has a major impact on the performance of the model because it directly influences the training process. Efficient hyperparameter selection can avoid overfitting, improve results, and form a generalized model. Data augmentation was performed on the original image datasets to expand the size of the training dataset (Shorten and Khoshgoftaar 2019). In order to optimize computational resources while increasing dataset size, we have applied five geometric image augmentation methods that preserve the semantic information on the training images using the Albumentations library (Buslaev et al. 2020). The augmentations applied were vertical flip, random rotate, horizontal flip, transpose, and grid distortion. These augmentations were selected on the basis of the most useful transformations for the structures of interest in this work which are the cytoplasm, nucleus, chromosomes, and mitochondria.

For the training of our WAE-Net model, we used the Adam optimizer with an initial learning rate of 0.0001. We reduced the learning rate by a factor of 4 when the validation loss has stopped decreasing for ten epochs. The mini-batch size was set to 1. Next, weight balancing was performed so that the model is not biased towards a specific segmentation class.

Loss functions play a critical part in effective CNN learning. In order to reduce the validation loss, a combination of focal and dice loss function was used for training (Jadon 2020). Focal loss addresses class imbalance by down-weighting the contribution of easy training examples and therefore enabling the model to focus more on learning hard examples (Wang et al. 2020). This is crucial to our work, as the segmentation classes in this work, such as chromosomes, mitochondria, cytoplasm, and nucleus have unbalanced representation in the image, thereby increasing the risk of the training being dominated by the most prevalent class. The mathematical definition of focal loss is shown in Eq. (1),

1 $$L_{fl} = - \alpha (1 - p)^{\gamma } \log (1 - p)$$

where, *p* is the model’s estimated probability, α, γ are two hyperparameters, α is used to adjust the distribution of the easy samples, and (1-*p*)^γ^ is the dynamic scaling factor, which is used to adjust the distribution of hard samples. 

The dice loss was introduced by Milletari et al. (2016) and is derived from the Sørensen–Dice coefficient. It is commonly used for bio-medical image segmentation tasks. Equation (2) shows the mathematical definition of dice coefficient, in which *p*_*i*_ and *g*_*i*_ represent pairs of corresponding pixel values of prediction and ground truth, respectively.

2 $$\begin{gathered} D = \frac{{2\sum\nolimits_{i}^{N} {p_{i} g_{i} } }}{{\sum\nolimits_{i}^{N} {p_{i}^{2} + \sum\nolimits_{i}^{N} {g_{i}^{2} } } }} \hfill \\ \hfill \\ \end{gathered}$$

### Weighted average ensemble training

**Fig. 4:**
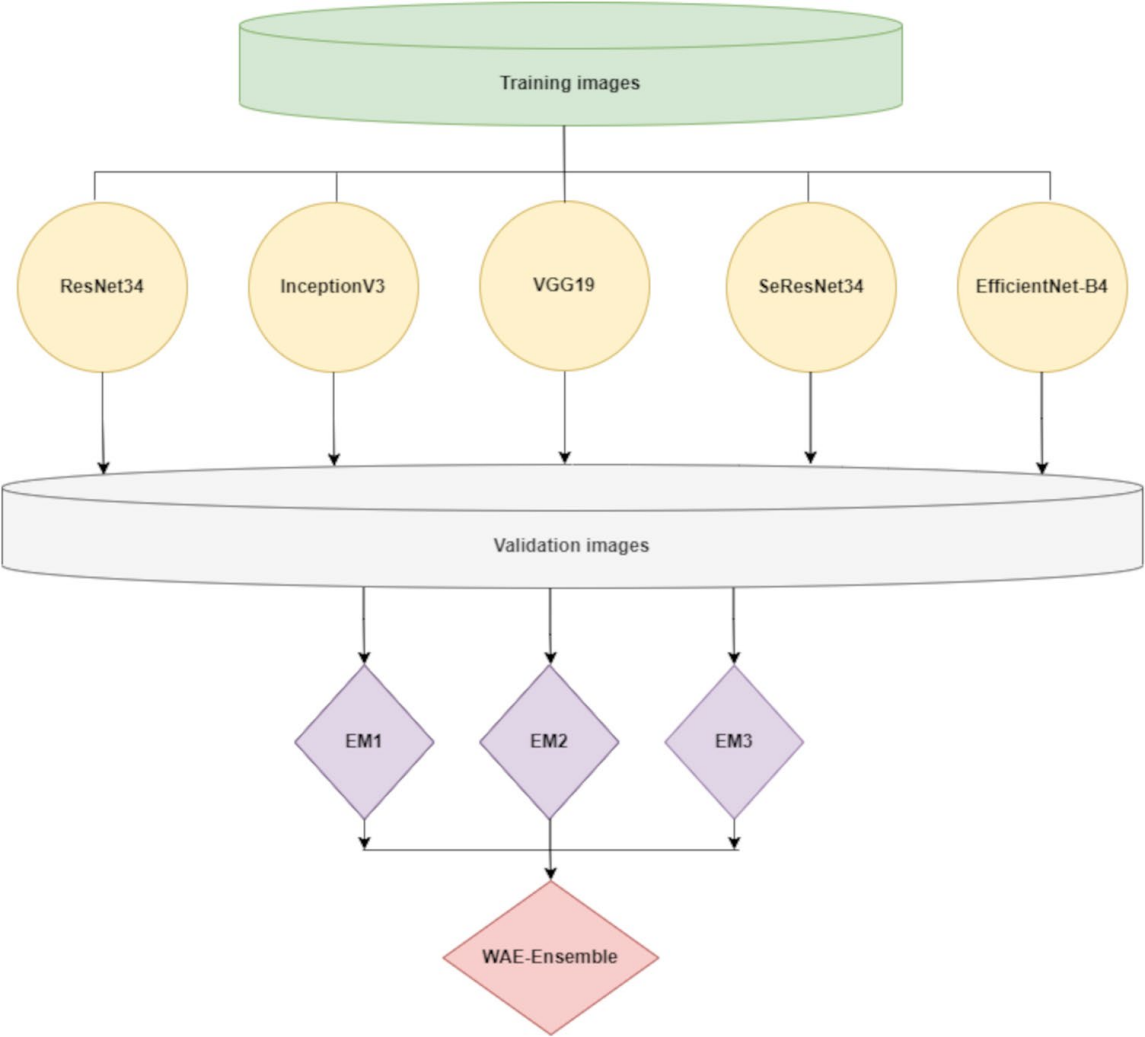
Graphical representation of the proposed WAE-Net. ResNet34, InceptionV3, VGG19, SeResNet34, and EfficientNet-B4 are the pre-trained networks that serve as the base-learners. EM1, EM2, and EM3 represent the predictions of the top-three best-performing base-learners that make up the ensemble. WAE-Net denotes the final prediction for our proposed ensemble

Our weighted average ensemble, WAE-Net, is constructed by combining the predictive base-learners, where the contribution of each base-learner to the final prediction is weighted by the performance of the individual learner. A representative diagram of the proposed WAE-Net is shown in Fig. 4.

We experimented with our ensemble method to segment the EM images into various biological classes such as cytoplasm, nucleus, mitochondria, and chromosomes. For each dataset, its top-three best-performing base-learners were combined together for the ensemble. We avoided including all base-learners because not all learners performed well. The inclusion of weak-performing base-learners in the ensemble wastes computational resources and time.

The challenging aspect of using a weighted average ensemble is how to choose the relative weighting for each ensemble member. We used the grid search method to search for the appropriate weights between 0 and 1 for each ensemble member based on the training data, where the learners with better performance receive a higher weight (Liashchynskyi and Liashchynskyi 2019). The best optimized weights are those that result in performance that is better than any contributing individual base-learner and an ensemble that uses equal weights. These weights are then multiplied by the prediction made by the individual base-learners and the weighted average is then used for the final prediction. The prediction of the ensemble was then tested on the test images. We use the original U-Net as a benchmark comparison for evaluating the performance of our WAE-Net. The same hyperparameters, losses, augmentations, and data split with cross-validation as described above were applied for both the U-Net and WAE-Net for the training process.

### Explainable segmentation using Grad-CAM

While CNN-based architectures yield impressive performance for image segmentation, their black-box nature makes it challenging to understand why it has given a specific prediction (Lin et al. 2019). Therefore, an interpretative and transparent model will be highly valuable to understand how the CNN makes its decisions, thereby allowing biologists to perform more informative and efficient data analysis.

In our effort to develop such models, we employ the Grad-CAM technique (Selvaraju et al. 2017) to visually show the activation regions of a particular biological class in EM images for model prediction. Grad-CAM exploits the spatial information that is preserved through convolutional layers of the CNN in order to understand which parts of an input image were important for the prediction. Grad-CAM specifically measures the gradients of features maps in the final convolution layer on a CNN model for an image to identify the critical regions that are class-discriminating saliency maps. The class-discriminative saliency map, $L^{c}$ for the target biological class, *c* in an image is defined as follows,

3 $$\begin{gathered} L_{i,j}^{c} = {\text{Re}} LU\left(\sum\limits_{k} {w_{k}^{c} } A_{i,j}^{k}\right ) \hfill \\ \hfill \\ \end{gathered}$$

where, *A*^*k*^_*i,j*_ denotes the activation map for the *k*-th filter at a spatial location *(i,j)* and Re*LU* captures the positive features of the target class. The target class weights of the *k*-th filter are computed as given by Eq. (4).

4 $$w_{k}^{c} = \frac{1}{Z}\sum\limits_{i} {\sum\limits_{j} {\frac{{\partial Y^{c} }}{{\partial A_{i,j}^{k} }}} }$$

The field-of-view (FOV) of a network is the size of the region in the input space that produces the feature. It is a measure of association of an output feature to the input region (Luo et al. 2017). The FOV plays an important role for Grad-CAM class-discriminating saliency maps because it gives an indication of where we are obtaining our results from as data flows through the layers of the network. The advantages of FOV in recognizing visual patterns lie in the fact that the units or neurons within a layer are directly tasked with learning visual features from a small region of the input data. It is therefore imperative to have a convolutional model with a FOV that covers the entire relevant input image region. The Grad-Cam takes an input image and predicts the output class-discriminating saliency map. However, if the network does not have the capacity to consider all the relevant pixels when performing the predictions, the resultant activation map will not be complete. Taking this into account, all the pre-trained networks used in this work have FOVs that cover the entire input image to yield correct information.

In our work, the Grad-CAM visualization is incorporated at the end edge of the proposed ensemble model after the training process, as displayed in Fig. 1. The GradCAM is applied to the convolutional layers at the U-Net bottleneck, which is at the end of the encoder before upsampling process. The class discriminatory saliency map for a specific biological class is then calculated and used to create an activation heat-map. The heat-map is then superimposed onto the given input image. The final Grad-CAM image enables us to verify that the proposed model obtained information of the biological structures from the correct regions in the images. It also enables us to identify the various regions in the EM image that are important for the prediction of each of the base-learners in the WAE-ensemble.

### Evaluation metrics

A successful segmentation by a CNN model is one that maximizes the overlap between the predicted and ground truth regions in an image. In order to evaluate the performance of the proposed method, we considered two commonly used evaluation metrics for semantic segmentation tasks, which are the Jaccard index and the F1 score. The Jaccard index (JI), which is also referred to as the Intersection over Union (IoU) metric, (Ronneberger et al. 2015; Cetina et al. 2018) quantifies the percentage of overlap between the ground truth mask and predicted output mask. The Jaccard index is defined by Eq. (5) below,

5 $$Jaccard\;index = \frac{GT \cap PO}{{GT \cup PO}}$$

where, *GT* and *PO* denote the pixels in the ground truth mask and predicted output respectively.

The F1 score (Gadosey et al. 2020) conveys the balance between the precision and the recall of the model and is defined by Eq. (6).

6 $$F1 - score = \frac{{2\left| {GT \cap PO} \right|}}{{\left| {GT} \right| + \left| {PO} \right|}}$$

Both of these metrics range from 0 to 1, with 0 signifying no overlap and 1 signifying a perfectly overlapping segmentation.

## Results

### Quantitative results

For each of the datasets, the five base-learners were trained and the top-three best-performing learners were then selected and combined together in order to construct the weighted average ensemble. The best-performing learners and their corresponding ensemble weighting for each dataset are shown in Table 4, whereby the sum of the weights for each dataset is equal to 1. Base-learners with zero weighting were not included in the ensemble. Based on our experiments (data not shown), grid search on different subsets of the training data based on the 5 × 5-fold cross validation lead to the same weighting for the top-three performing models for each dataset.

**Table 4:** Top-three best-performing base-learners for each dataset and their corresponding ensemble weighting obtained from Grid-CV search

Dataset	Top-three best-performing base-learners	Weighting
Dataset 1	ResNet34, InceptionV3, VGG19	0.34, 0.33, 0.33
Dataset 2	ResNet34, InceptionV3, EfficientNet-B4	0.50, 0.50, 0.00
Dataset 3	ResNet34, SeResNet34, EfficientNet-B4	1.00, 0.00, 0.00
Dataset 4	ResNet34, VGG19, EfficientNet-B4	0.50, 0.00, 0.50
Dataset 5	ResNet34, InceptionV3, EfficientNet-B4	0.40, 0.40, 0.20
Dataset 6	ResNet34, InceptionV3, EfficientNet-B4	0.40, 0.20, 0.40
Dataset 7	ResNet34, InceptionV3, VGG19	0.40, 0.20, 0.40

ResNet34 has emerged as the best-performing base-learner for all the seven EM datasets. This was followed by InceptionV3 and EfficientNet-B4, respectively. SeResNet34 was the most poorly performing base-learner, as it only emerged once as a top-three best performer. In terms of ensemble weighting, the combination of two or more base-learners resulted in the best performance for most of the datasets. However, dataset 3 did not benefit from an ensemble model as only the contribution of ResNet34 was useful for its final prediction (Table 4).

After the construction of the WAE-Net using the best-performing base-learners, we evaluated its segmentation performance and compared it with the U-Net (Table 5). The results were quantitatively evaluated by using the Jaccard index and F1 score metric. WAE-Net yielded a mean Jaccard index (JI) of 0.9087 and mean F1 score of 0.9203 for all the seven datasets while U-Net only yielded a mean JI of 0.7709 and mean F1 score of 0.7680.

**Table 5:** Segmentation performance comparison between WAE-Net and U-Net

	U-Net		WAE-Net	
	Jaccard index	F1 score	Jaccard index	F1 score
Dataset 1	0.8552	0.8980	**0.9865**	**0.9923**
Dataset 2	0.7645	0.6942	**0.8448**	**0.8842**
Dataset 3	0.5571	0.5381	**0.7243**	**0.6915**
Dataset 4	0.7686	0.7866	**0.9692**	**0.9685**
Dataset 5	0.8753	0.7994	**0.9725**	**0.9784**
Dataset 6	0.8456	0.7924	**0.9644**	**0.9817**
Dataset 7	0.7099	0.8676	**0.8992**	**0.9452**

The Jaccard index and F1 score were averaged over all test images in each dataset

**Fig. 5:**
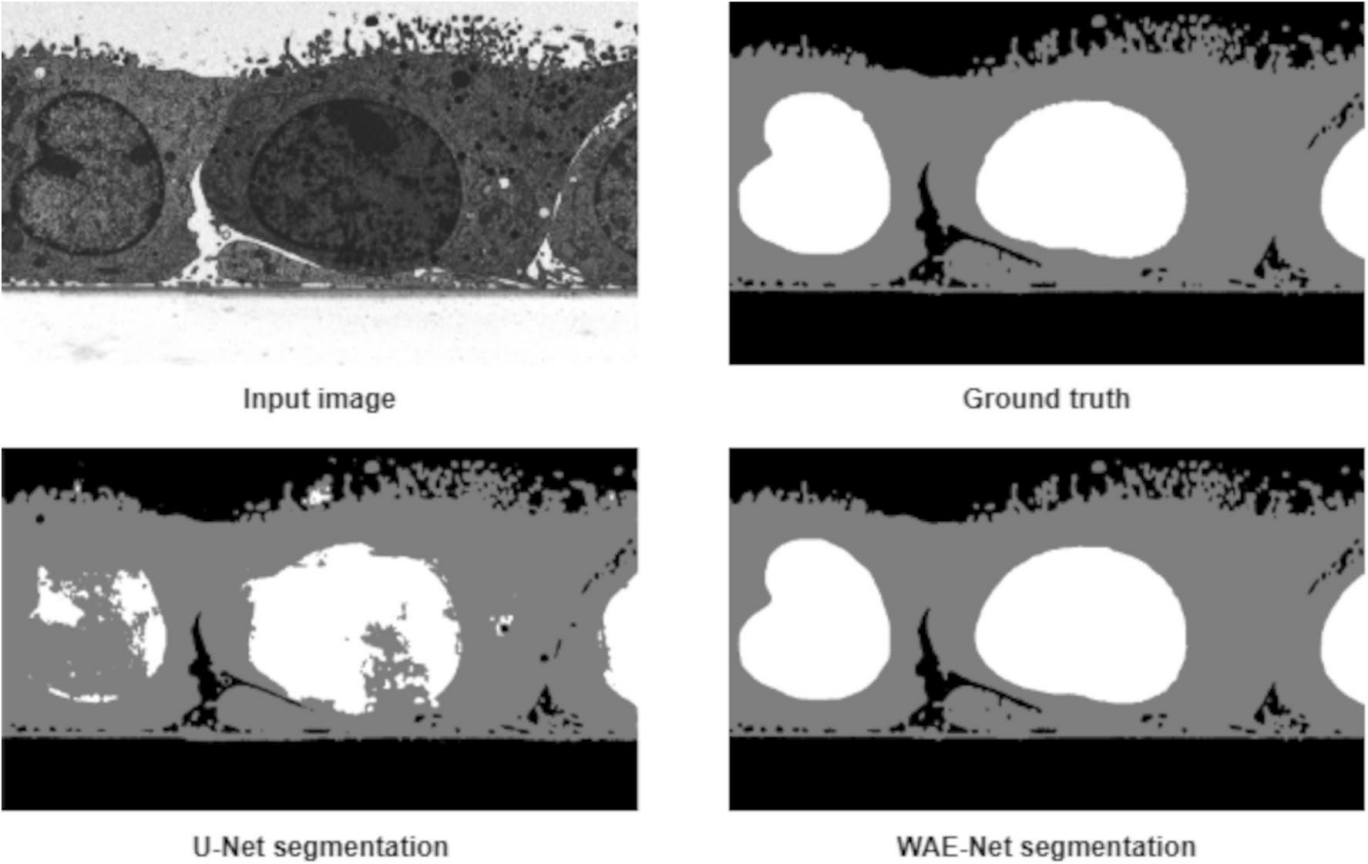
Quantitative evaluation of the segmentation results produced by WAE-Net and U-Net over a single image in dataset 1. The ground truth and predicted output images contains three segmented structures which are the background (black), cytoplasm (grey), and nucleus (white)

**Fig. 6:**
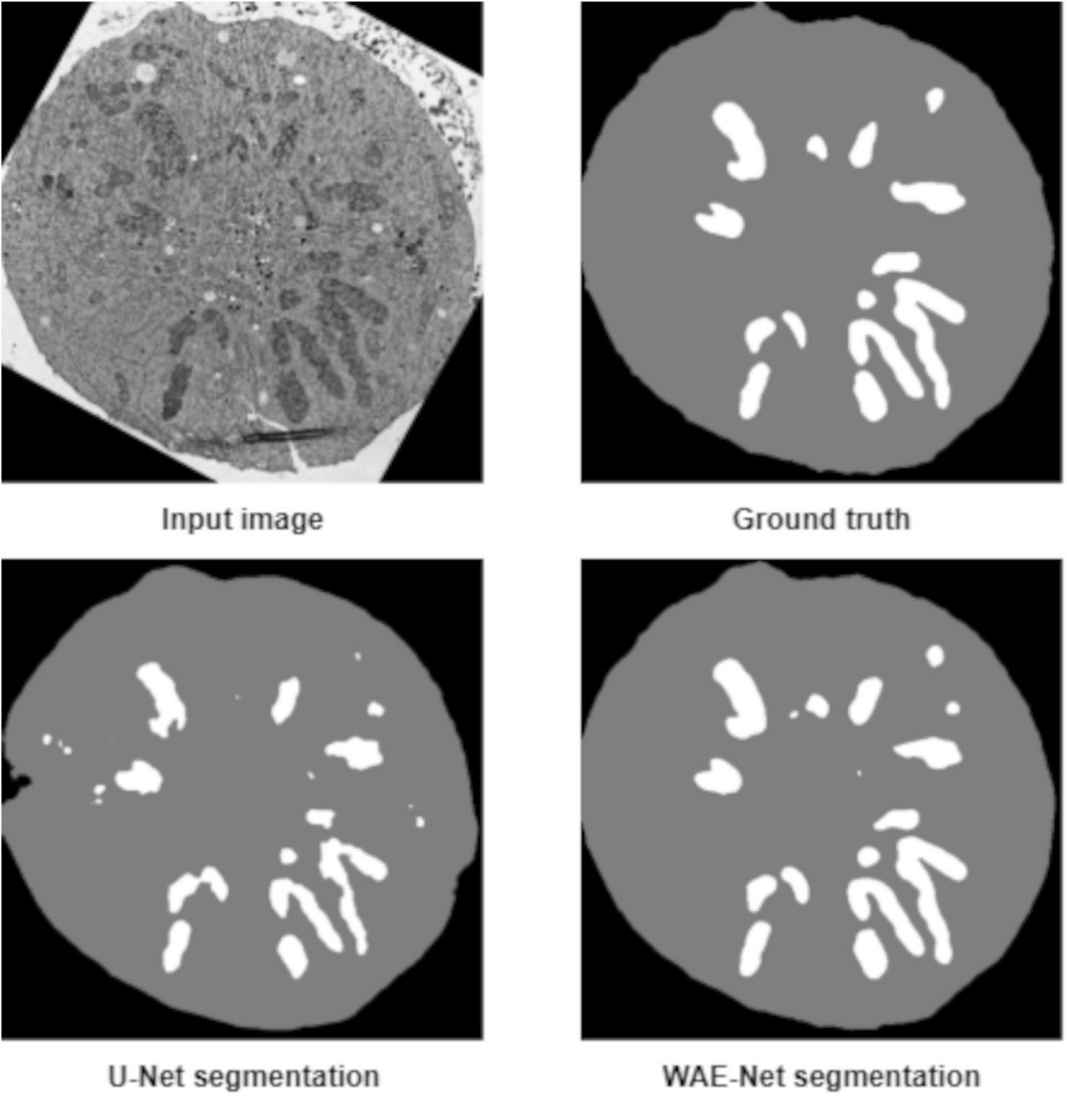
Quantitative evaluation of the segmentation results produced by WAE-Net and U-Net over a single image in dataset 2. The ground truth and predicted output images contains three segmented structures which are the background (black), cytoplasm (grey), and chromosomes (white)

**Fig. 7:**
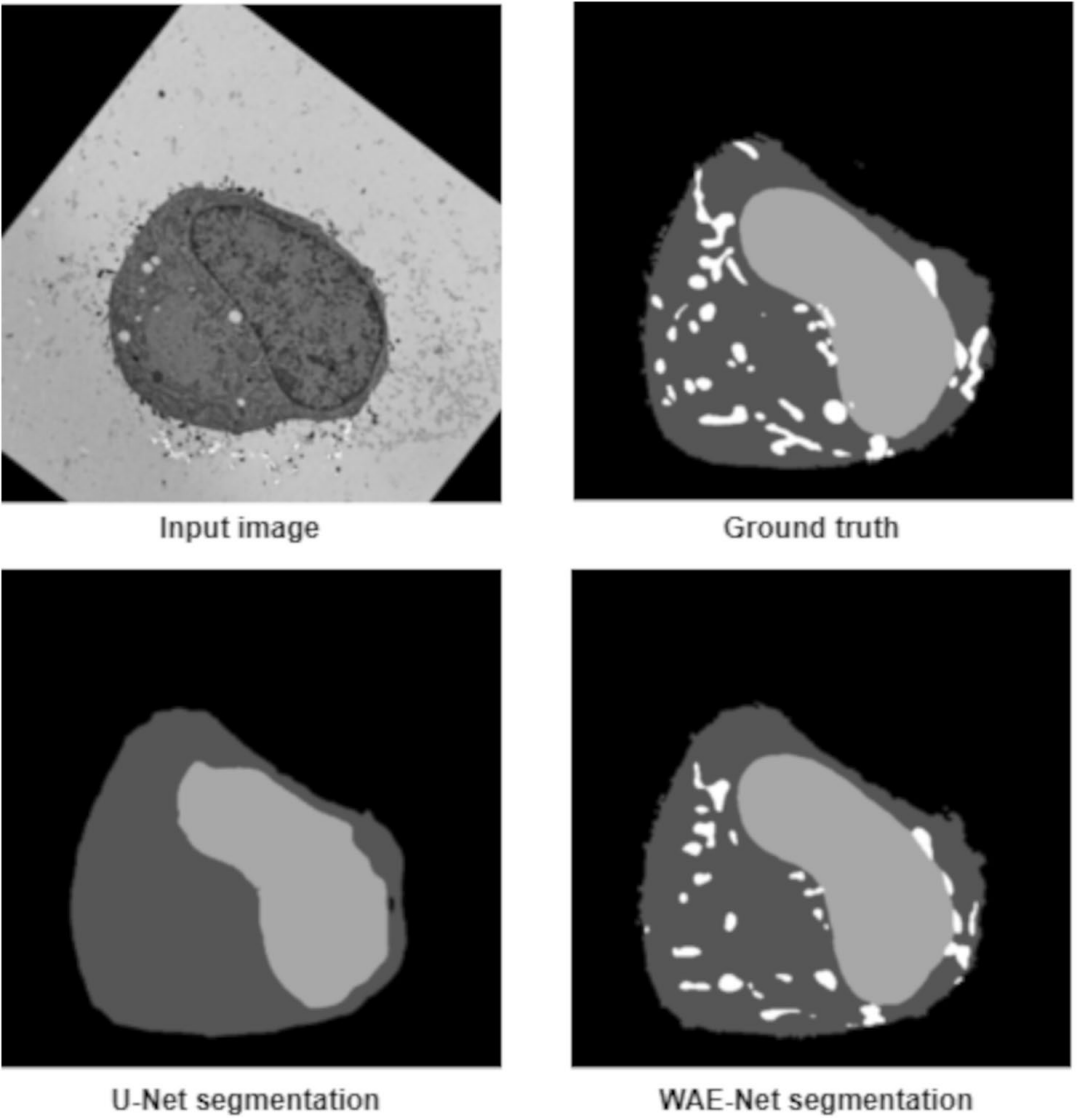
Quantitative evaluation of the segmentation results produced by WAE-Net and U-Net over a single image in dataset 3. The ground truth and predicted output images contains four segmented structures which are the background (black), cytoplasm (dark grey), nucleus (light grey) and mitochondria (white)

In order to further understand the segmentation results obtained by the proposed model, we evaluated the performance of both WAE-Net and U-Net on the individual segmentation classes (Table 6). Both U-Net and WAE-Net were able to segment the background class similar to ground truth data with a mean JI of above 0.90 for all datasets. Our model performed very well in segmenting the cytoplasm with a mean JI of 0.9160 while U-Net’s performance deteriorated with a mean JI value of 0.7636. When segmenting the nucleus, our ensemble model yielded good segmentation results with a mean JI of 0.9309 while the U-Net had only managed to achieve a mean JI of 0.6966. The chromosomes were able to be segmented by WAE-Net with a JI of 0.6826 compared to U-Net which performed poorly with a JI of only 0.4867. Both models performed very badly for the segmentation of mitochondria in dataset 3. The U-Net was not able to segment any mitochondria at all, while our model was able to segment only some of the mitochondria pixels with a JI of 0.2143. The reason for the low segmentation rates for both chromosomes and mitochondria might be because both of these structures are quite small compared to the other structures in the EM images and therefore our model did not have sufficient pixel coverage in the training images for learning. Furthermore, in dataset 3, there were very few training images but a higher number of segmentation classes which explains the low segmentation rate for chromosomes. Generally, WAE-Net was able to consistently obtain superior performance compared to the U-Net for all datasets as well as for all individual segmentation classes (Tables 5 and 6, Figs. 5, 6, 7 and S1). Dataset 1 had the best performance while the worst was from dataset 3.

**Table 6:** Segmentation performance comparison between WAE-Net and U-Net for each segmentation class

Dataset	Segmentation class	U-Net	WAE-Net
Dataset 1	Background	0.9296	**0.9567**
	Cytoplasm	0.8663	**0.9882**
	Nucleus	0.7742	**0.9918**
Dataset 2	Background	0.9157	**0.9321**
	Cytoplasm	0.8936	**0.9143**
	Chromosomes	0.4867	**0.6826**
Dataset 3	Background	0.9547	**0.9762**
	Cytoplasm	0.6858	**0.8416**
	Nucleus	0.5783	**0.8052**
	Mitochondria	0.0000	**0.2143**
Dataset 4	Background	0.9344	**0.9897**
	Cytoplasm	0.6838	**0.9464**
	Nucleus	0.6728	**0.9676**
Dataset 5	Background	0.9646	**0.9912**
	Cytoplasm	0.8166	**0.9452**
	Nucleus	0.8372	**0.9645**
Dataset 6	Background	0.9769	**0.9941**
	Cytoplasm	0.8154	**0.9567**
	Nucleus	0.7461	**0.9569**
Dataset 7	Background	0.9562	**0.9765**
	Cytoplasm	0.5838	**0.8191**
	Nucleus	0.5712	**0.8994**

The performances are evaluated in terms of Jaccard index

We also performed a small experiment to investigate the performance of smaller base-learner U-Nets compared to their relatively larger counterparts that were used in our WAE-Net. The size was defined by the number of learning parameters and convolutional layers and the performance was evaluated using Jaccard index (JI).

We compared the performance of base-learners using ResNet18 and ResNet34, VGG16 with VGG19, and EfficientNetB0 with EfficientNetB4. Both ResNet and VGG were trained on a subset of data from dataset 1 while EfficientNet was trained on a subset of data from dataset 4. We observed that ResNet18, which has 18 convolutional layers and 14,340,860 parameters, gave a Jaccard index (JI) of 0.8045 while ResNet34 with 34 layers and 24,456,444 parameters gave a JI of 0.8288. VGG16 that has 16 layers and 23,752,563 parameters yielded a JI of 0.8147 while VGG19 with 19 layers and 29,062,259 parameters yielded a JI of 0.8197. We compared EfficientNetB0, which has 10,115,791 parameters with EfficientNetB4, which has 25,735,307 parameters and observed that the former had a JI of 0.7622 while the latter had a JI of 0.8206. This indicates that the smaller networks performed almost as well as their relatively larger counterpart for the structures of interest in this work, with the larger counterparts giving only slightly improved performances. Surprisingly, VGG16 and VGG19 gave almost similar performance.

### Qualitative results

Figures 5, 6, 7, and S1 show representative test images after segmentation, comparing input image, ground truth, WAE-Net prediction, and U-Net prediction. An expert biologist verified that the WAE-Net was able to segment the biological structures in all test EM images more accurately compared to U-Net. Both the models were able to segment the background very well, however, the U-Net was not able to segment the boundary of both cytoplasm and nucleus accurately and was only able to detect the chromosomes partially. In comparison, our ensemble model was able to segment the cytoplasm and nucleus in a highly accurate manner and was able to detect most of the chromosomes. We observed that our model detected some of the mitochondria present in dataset 2 (Fig. 6) and classified them as chromosomes. This could be attributed to the fact that the chromosomes and mitochondria shared certain similarities in terms of appearance and there were very small amounts of mitochondria present in dataset 2 for our model to learn to differentiate between these two structures.

The worst performance was obtained from dataset 3 (Fig. 7), where both models were unsuccessful in segmenting the mitochondria. Our model was not able to segment the mitochondria in the images in a manner similar to the ground truth. Most of the mitochondria were not detected. However, the ensemble model still exhibited better performance, as the U-Net was not able to detect any mitochondria at all and was incorrect in segmenting the cytoplasm and nucleus. Overall, based on the segmented images obtained, we are able to visually verify that our model was able to give segmentation results that are nearer to the ground truth compared to the U-Net.

### Visualization with Grad-CAM

**Fig. 8:**
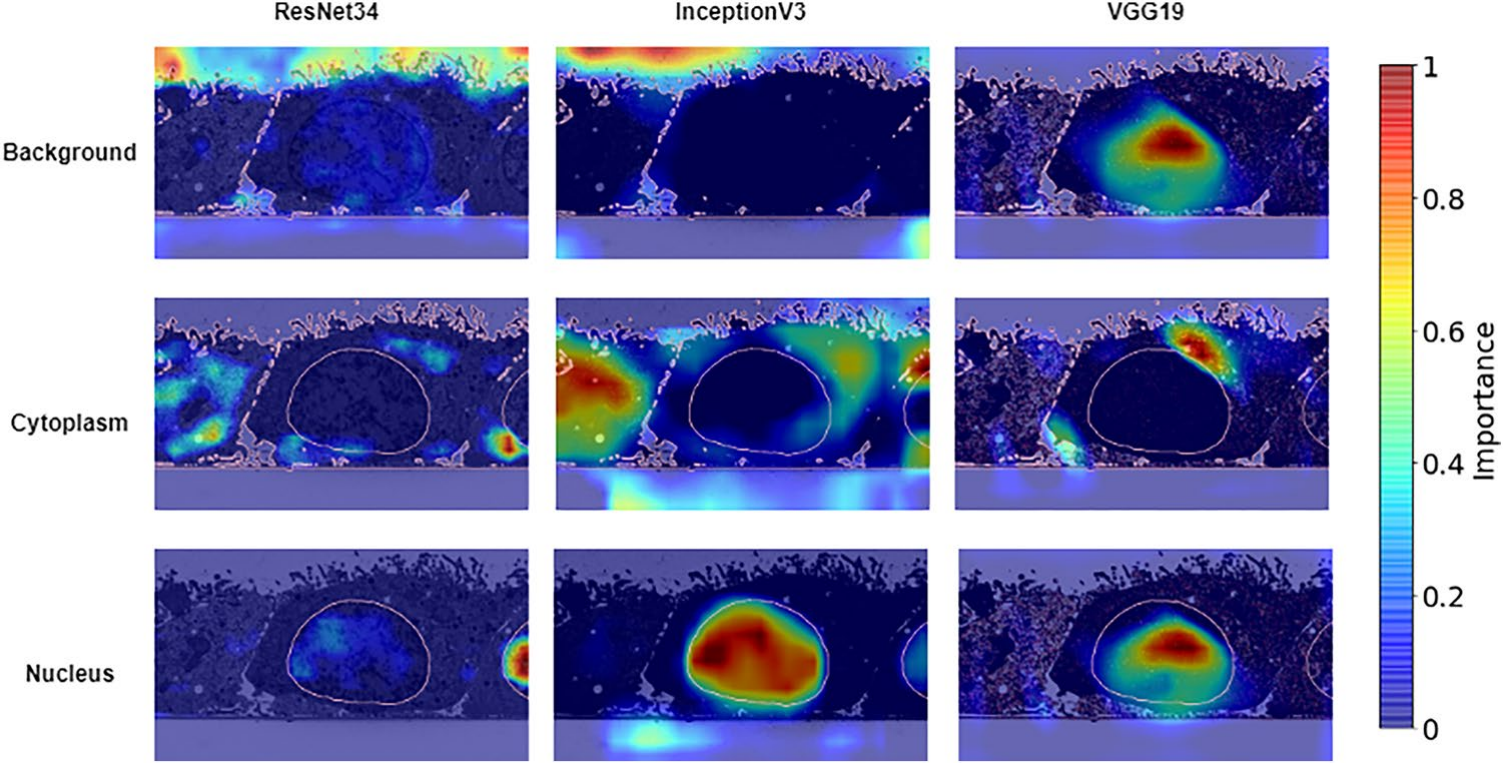
Grad-CAM visualization on the input image of Fig. 5 using the WAE-Net. These visualizations were obtained from the bottleneck layer of the base-learners of our network. The columns indicate the respective base-learners while the rows indicate the target segmentation class localized by Grad-CAM. The red regions highlight the most important discriminative regions, while the blue regions the least important. The pink outline indicates that the region of interest is contained within this area

We applied the Grad-CAM method to visually depict the pertinent areas in the test images where WAE-Net emphasizes the segmentation decision for a given biological class. We found that the convolutional layers of the U-Net bottleneck, which is at the end of the encoder before upsampling, are more informative than layers close to the end of the U-Net decoder. Figure 8 and S2 show representative images of the Grad-CAM visualization using the top-three base-learners for each segmentation class. The Grad-CAM visualization locates the relevant areas in the EM image that is important for the segmentation of a specific biological structure for each of the base-learners. The red regions highlight the most important discriminative regions and the blue regions the least important as depicted by the color bar in Fig. 8.

Importantly, we were able to learn the behavior of the individual base-learners that compose the WAE-Net. It was observed that not all base-learners in the WAE-Net were triggered by the same region for prediction. Different base-learners look at different areas in an image for the segmentation of the same biological structure. Figure 8 shows that both ResNet34 and InceptionV3 were triggered by the correct regions to identify the background of the EM image. However, VGG19 was triggered by regions that were outside of the background area, specifically the nucleus for background segmentation. For the cytoplasm, all the three base-learners were triggered by the correct region. GradCAM visualizations indicate that different areas within the cytoplasm was important for different base-learners. In example, the cytoplasm area that was very relevant for InceptionV3 prediction was however less relevant for ResNet34 and VGG19. Both InceptionV3 and VGG19 were strongly triggered by the main nucleus in the image for nucleus segmentation. In contrary, ResNet34 was not triggered by the main nucleus, but rather by a small nucleus region at the right of the image. Further visualizations in S2 provide insight into the behavior of the individual base-learners. We observed in both Fig. 8 and S2 that areas that were not part of the object of interest were also triggered as relevant by the model for prediction.

## Discussion

The key to new insights and discoveries about cell structures and organelles goes hand in hand with new technological developments that enable acquiring and analyzing relevant, high-quality information from EM images. However, the implementation of state-of-the-art DL approaches for biological analysis is often hindered by the requirement of large labeled training datasets, which are usually scarce in specialized fields such as biological EM. In order to provide a novel contribution towards eliminating this limitation, we have presented a robust approach for the fully automatic semantic segmentation of biological structures in EM images using limited training dataset. This study demonstrates that a weighted average ensemble of CNN models can significantly improve the segmentation rate compared to a single model trained with a small labeled dataset. We show the potential of this approach to segment both TEM and SEM.

We first constructed the individual base-learners using a transfer learning approach. This approach increased the segmentation ability of the learners significantly even though our images differ greatly from the ImageNet database. The base-learners were able to extract feature representations from ImageNet and successfully applied them to the EM images. Then, we combined the best-performing top-three learners for each dataset in a weighted average manner for the final segmentation.

We observed that the best base-learners differs for each dataset. This indicates that no one ultimate combination of base-learners gave the best results for all the datasets. This is because the datasets differ from one another in terms of image properties as well as biological appearances. Therefore, one base-learner might be suited to learn the semantic information of specific biological structure while another is better at a different one. Thereby, combining these base-learners in an ensemble model enables us to harness this learning power.

We have demonstrated that an ensemble model is imperative for a good segmentation. Most of the datasets gave the best results with a combination of two or more base-learners. Datasets 1, 2, and 4 had equal weighting among its ensemble members while, the rest of the datasets had unequal weighting (Table 4). This proves that not all base-learners contribute equally towards the performance of an ensemble model and weighting their contribution has a powerful effect on the final prediction. Only dataset 3 did not benefit from the ensemble process as only one base-learner significantly contributed to the final prediction. This is because this dataset contained a higher number of segmentation classes compared to the other datasets but was trained with comparatively low amount of training images (Table 2). Therefore, most of the base-learners were not able learn sufficient discriminative features for segmentation, rendering the ensemble model inefficient for this dataset.

The WAE-Net’s results were compared with the original U-Net from quantitative and qualitative perspectives. Our ensemble model outperformed the U-Net for all the datasets significantly (Tables 5 and 6 and Figs. 5, 6, 7 and S1). This suggests that an ensemble of models can outperform one single model when trained with only a small number of ground truth images. In addition, we investigated the performance of both the models on the individual segmentation classes (Table 6). Large differences between the two models were observed in their abilities to detect cytoplasm, nucleus, chromosomes, and mitochondria. Overall, the WAE-Net was able to segment the EM images in a manner similar to the ground truth for most of the datasets despite the low availability of training images. Our proposed model was able to generalize well on the hold-out test dataset (Figs. 5, 6, 7, and S1). When we look at the training time of the WAE-Net, it takes approximately between 4 and 8 h on a single GPU, depending on the pre-trained network parameters. Because it takes several months for a biologist to segment a large dataset manually, the total cost of training time is far less.

From the small study conducted to observe the performance of smaller pre-trained U-Net base-learners compared to their relatively larger counterparts, we learned that smaller networks than those utilized in this work are able to give good predictive performance. However, further study needs to be conducted with a larger variety of biological structures and electron microscopy modalities to understand the feasibility of small pre-trained U-Nets for semantic segmentation.

The Grad-CAM has demonstrated to be a powerful tool to visually understand how the ensemble model achieved the segmentation prediction obtained in this work (Figs. 8 and S2). Its inclusion enabled us to understand the behavior of the individual base-learners for the various segmentation classes. We observed that different regions were found relevant by different base-learners for the segmentation of the same structure. Since the WAE-Net model is a combination of learners, strongly relevant regions found by all learners were combined for the final prediction, thereby improving the model’s overall performance.

While Grad-CAM visualizations are class-discriminative and are able to localize relevant image regions well, they lack the ability to show fine-grained pixel-wise details. Despite this, we believe that the Grad-CAM based visualizations is a first step towards the incorporation of transparency and interpretability in deep learning for biological EM applications. As deep learning is a black-box approach in the computer science domain, the inclusion of these visualizations may increase biologists trust in the model’s prediction. It may also be useful for future applications where model selection is crucial.

Concludingly, this work explored the possibility of semantically segmenting EM images using a very small training dataset. Our model has shown the ability to segment four types of biological structures in both TEM and SEM images. More importantly, the WAE-Net segmentation predictions provide a close estimate of the ground truth data. The standard U-Net might be able to reach and even surpass the performance of WAE-Net given the availability of large amount of labeled ground truth data. However, since the availability of large amounts of labeled data is often scarce in the biological EM field, we believe that our approach can lead to the rapid development of deep learning applications in this area.

Since our method reduces the labeling burden on biologists, it could be further adopted for the segmentation of various other structures in the field of biological image analysis, as well as for other types of microscopy modalities. Apart from that, our approach can also be used with many other pre-trained networks not explored in this work. Therefore, WAE-Net can be easily customized to suit the individual segmentation needs of the biologists. As there is still room for improvement in terms of segmentation performance for very small datasets, further pursuit in the direction of this study would be worthwhile. Finally, we believe that the integration of this tool into the morphological analysis of images could help biologists to have a deeper understanding of disease mechanisms as well as help expand the impact of artificial intelligence in the biological domain.

## Supplementary Information

Below is the link to the Electronic Supplementary Material.

Supplementary file1 (TIF 120989 KB)

Supplementary file2 (TIF 48727 KB)

Supplementary file3 (TIF 15948 KB)

Supplementary file4 (TIF 15948 KB)

Supplementary file5 (TIF 121102 KB)

Supplementary file6 (TIF 56219 KB)

Supplementary file7 (TIF 64030 KB)

Supplementary file8 (TIF 48727 KB)

Supplementary file9 (TIF 100986 KB)

Supplementary file10 (TIF 64030 KB)

Supplementary file11 (TIF 48727 KB)

Supplementary file12 (TIF 15948 KB)

Supplementary file13 (TIF 64030 KB)

Supplementary file14 (TIF 120989 KB)

Supplementary file15 (TIF 52223 KB)

Supplementary file16 (TIF 64030 KB)

Supplementary file17 (TIF 123356 KB)

Supplementary file18 (TIF 55886 KB)

Supplementary file19 (TIF 126624 KB)

Supplementary file20 (TIF 55886 KB)

Supplementary file21 (TIF 15948 KB)

Supplementary file22 (PDF 592 KB)

Supplementary file23 (PDF 1198 KB)

## Data Availability

The source codes, pre-trained weights and images used in this work can be accessed at https://data.mendeley.com/datasets/9rdmnn2x4x/1.
