# Supplementary figures and images for: Weighted average ensemble-based semantic segmentation in biological electron microscopy images
Source: Histochem Cell Biol. 2022 Aug 20;158(5):447–62. doi: 10.1007/s00418-022-02148-3 (PMC9630254; doi:10.1007/s00418-022-02148-3)

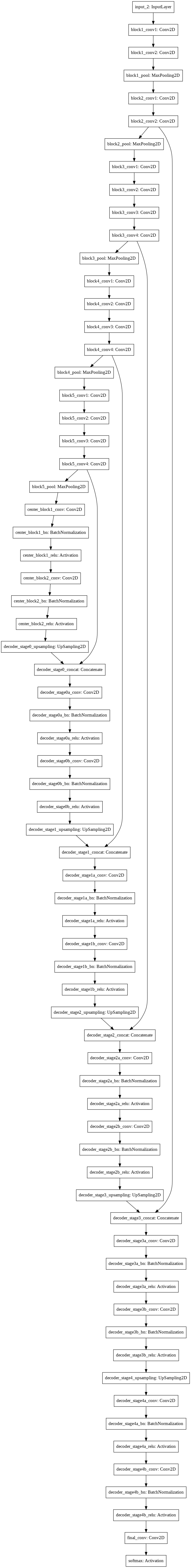

Supplement: Supplementary file 3 — Supplementary file3 (TIF 15948 KB) [file 418_2022_2148_MOESM3_ESM.tif]

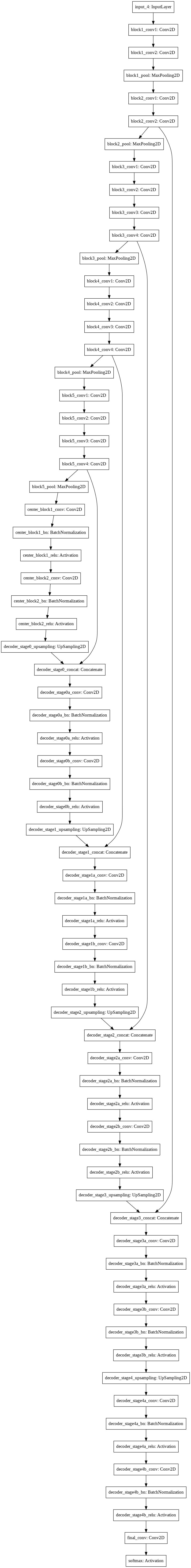

Supplement: Supplementary file 4 — Supplementary file4 (TIF 15948 KB) [file 418_2022_2148_MOESM4_ESM.tif]

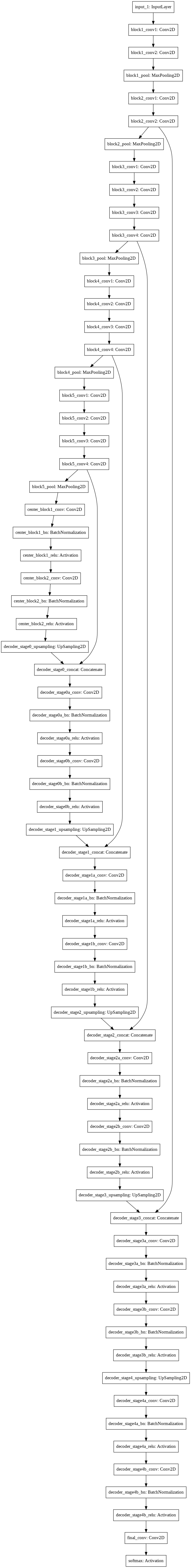

Supplement: Supplementary file 12 — Supplementary file12 (TIF 15948 KB) [file 418_2022_2148_MOESM12_ESM.tif]

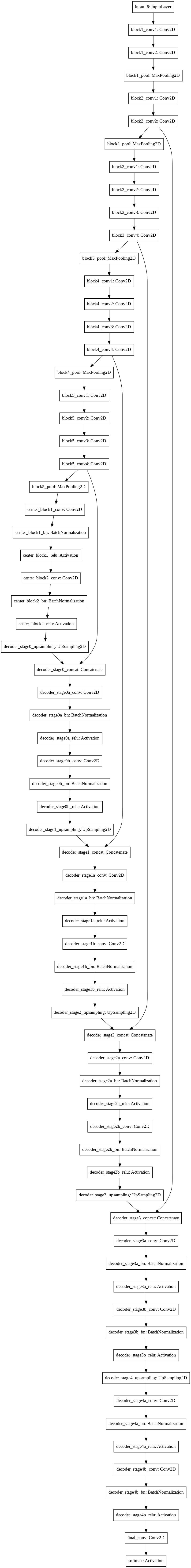

Supplement: Supplementary file 21 — Supplementary file21 (TIF 15948 KB) [file 418_2022_2148_MOESM21_ESM.tif]
