# Supplementary material for: Weighted average ensemble-based semantic segmentation in biological electron microscopy images
Source: Histochem Cell Biol. 2022 Aug 20;158(5):447–62. doi: 10.1007/s00418-022-02148-3 (PMC9630254; doi:10.1007/s00418-022-02148-3)
Supplement: Supplementary file 22 — Supplementary file22 (PDF 592 KB) [file 418_2022_2148_MOESM22_ESM.pdf]

Supplementary Figure S1

Representative test image from dataset 4. Bar 2µm

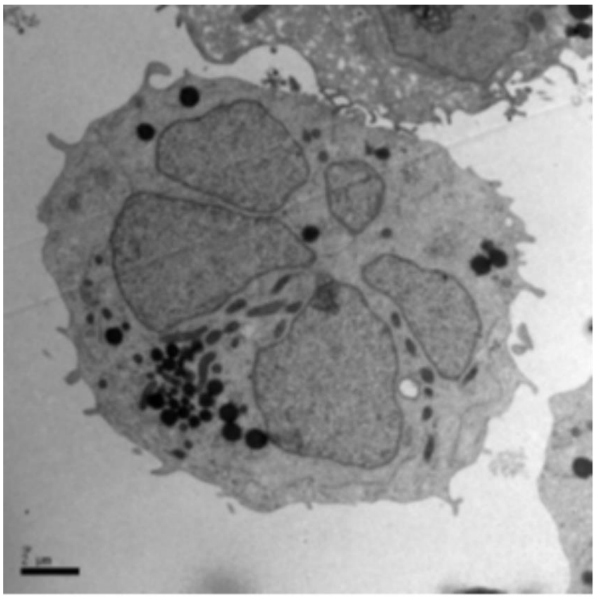

Input image

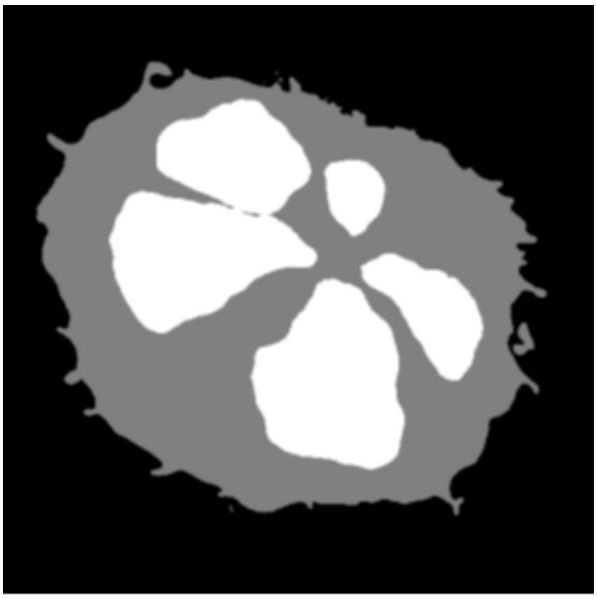

Ground truth

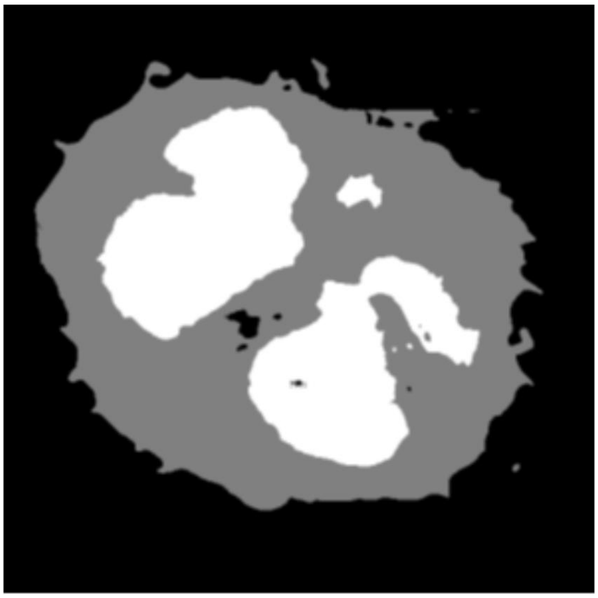

U-Net segmentation

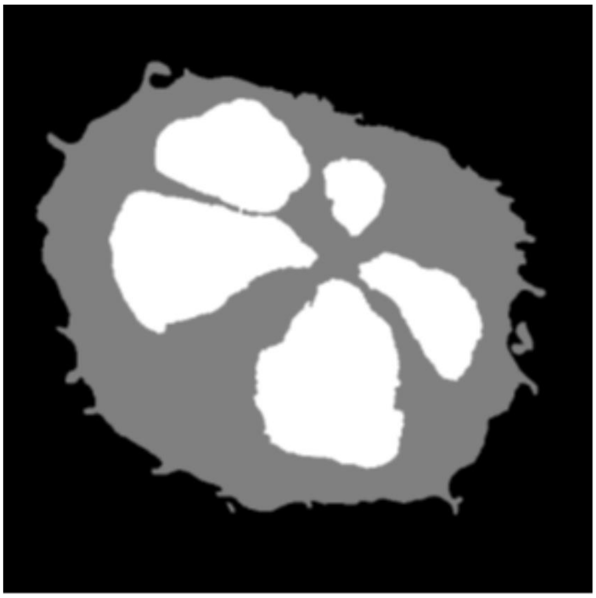

WAE-Net segmentation

Representative test image from dataset 5. Bar 2µm

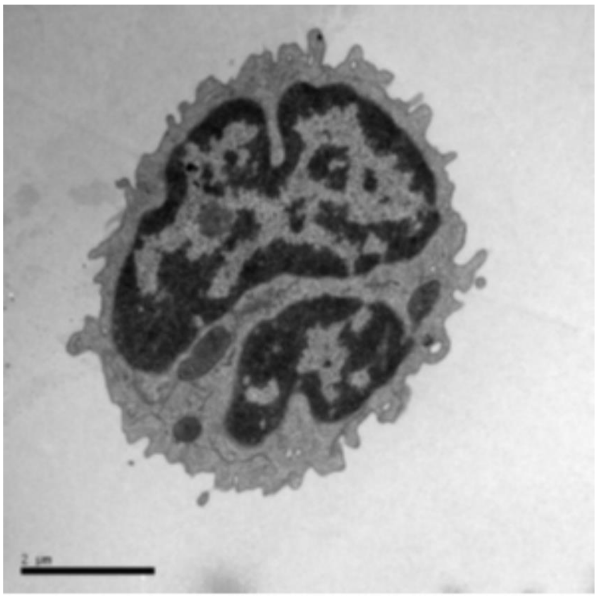

Input image

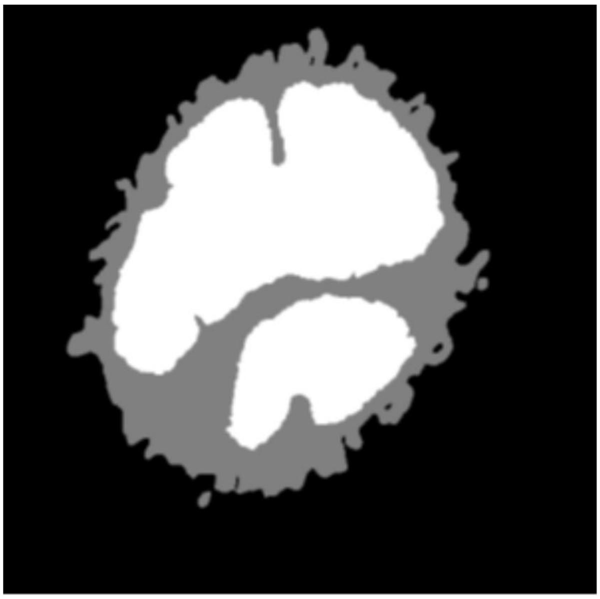

Ground truth

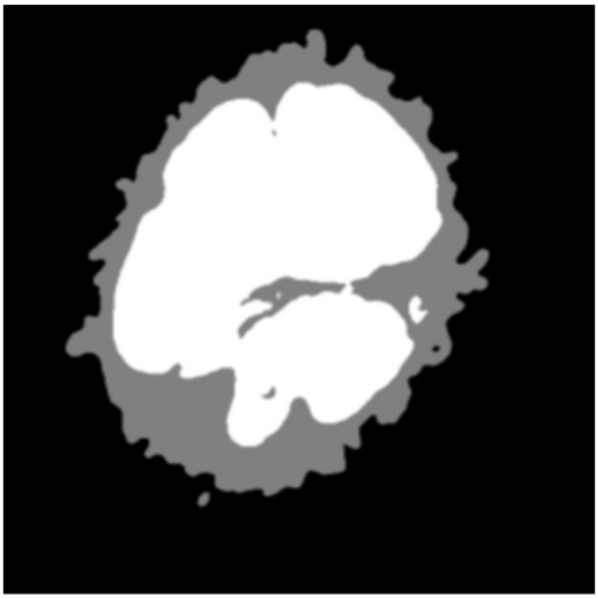

U-Net segmentation

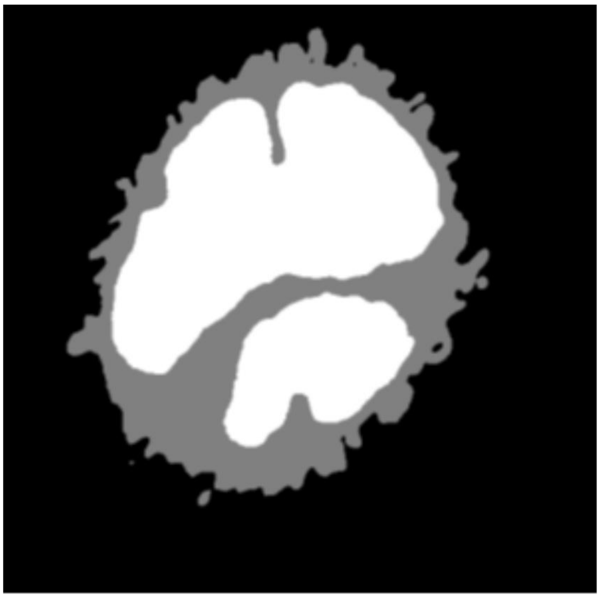

WAE-Net segmentation

Representative test image from dataset 6. Bar 2µm

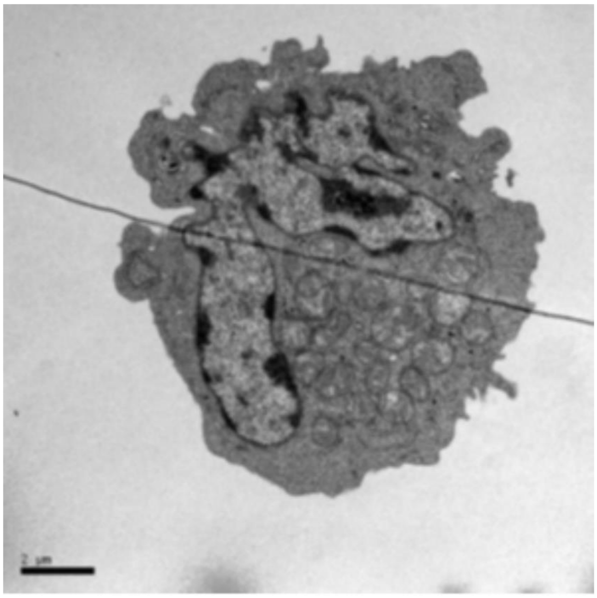

Input image

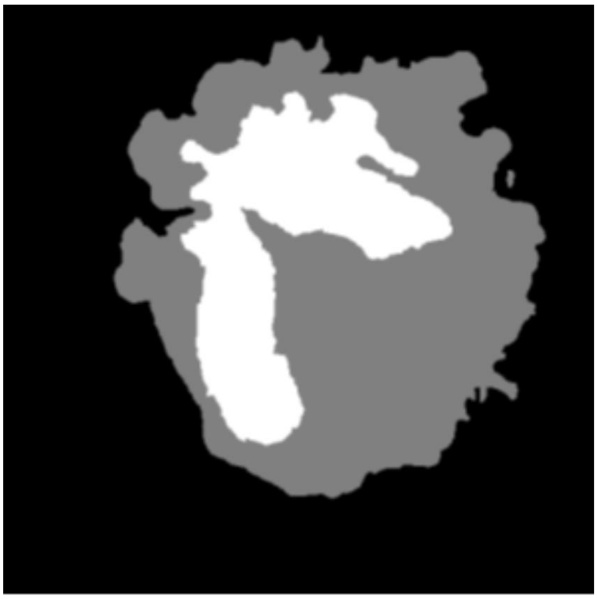

Ground truth

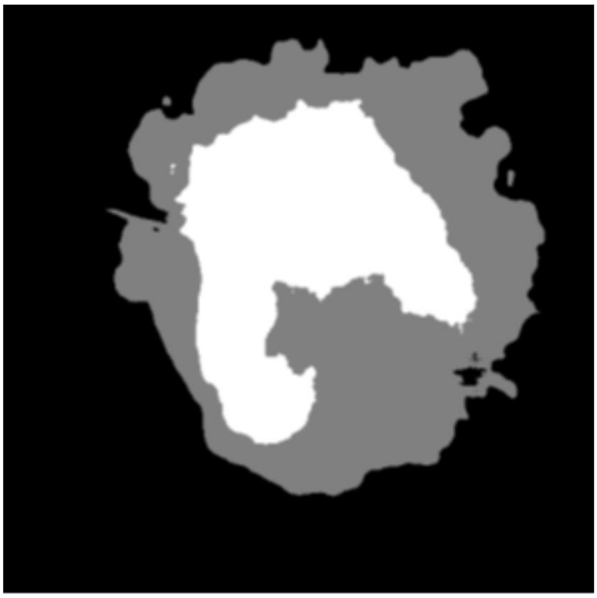

U-Net segmentation

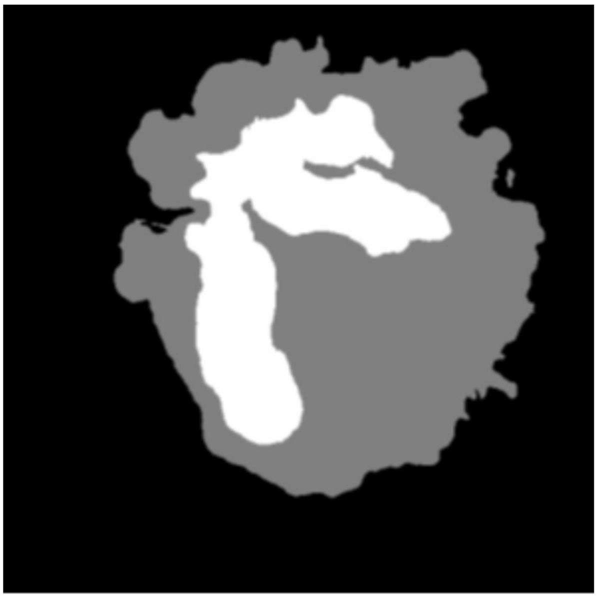

WAE-Net segmentation

Representative test image from dataset 7. Bar 2µm

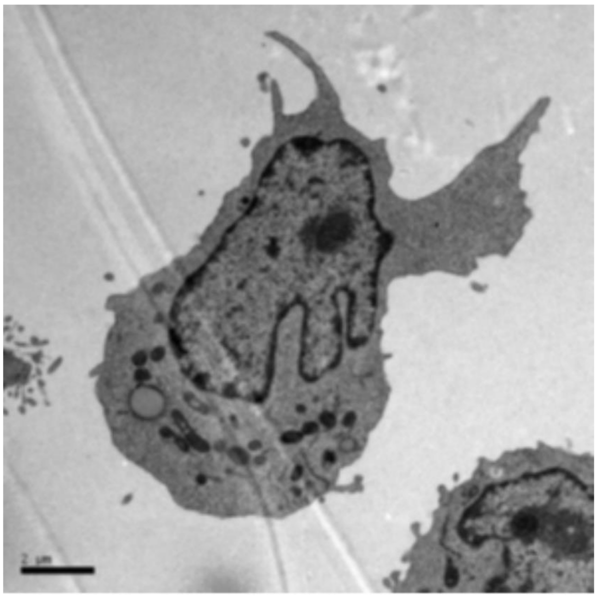

Input image

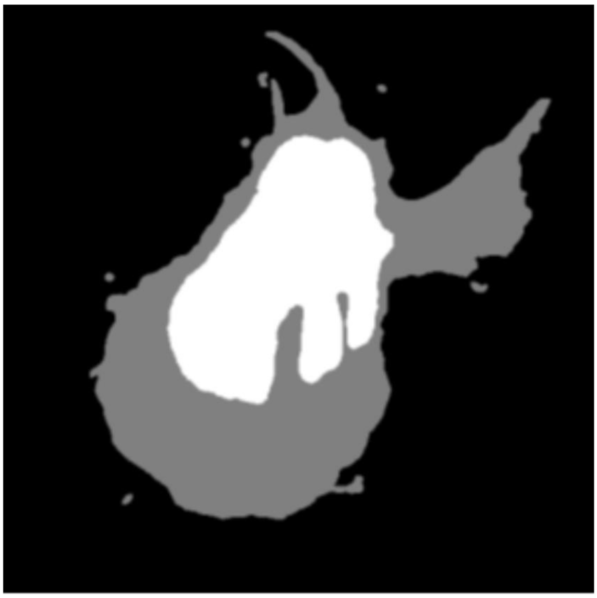

Ground truth

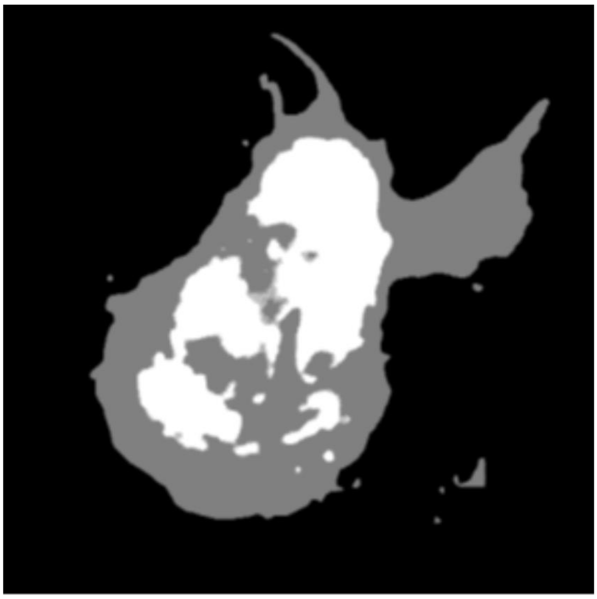

U-Net segmentation

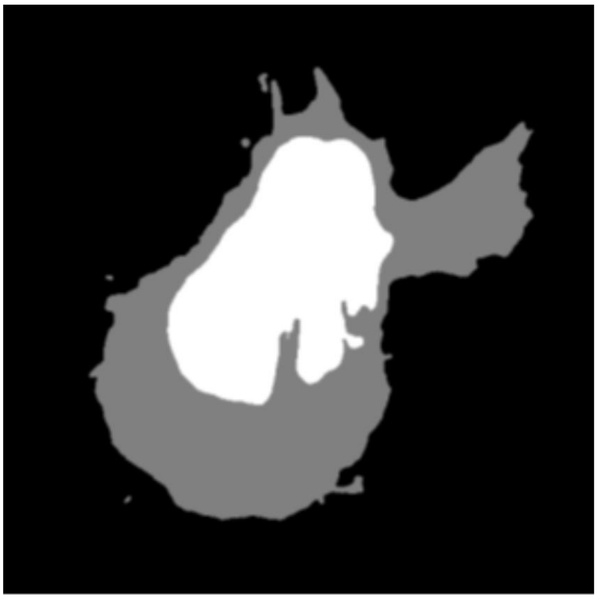

WAE-Net segmentation
