# Supplementary material for: Weighted average ensemble-based semantic segmentation in biological electron microscopy images
Source: Histochem Cell Biol. 2022 Aug 20;158(5):447–62. doi: 10.1007/s00418-022-02148-3 (PMC9630254; doi:10.1007/s00418-022-02148-3)

Supplementary Figure S2

Representative Grad-CAM images from Dataset 2

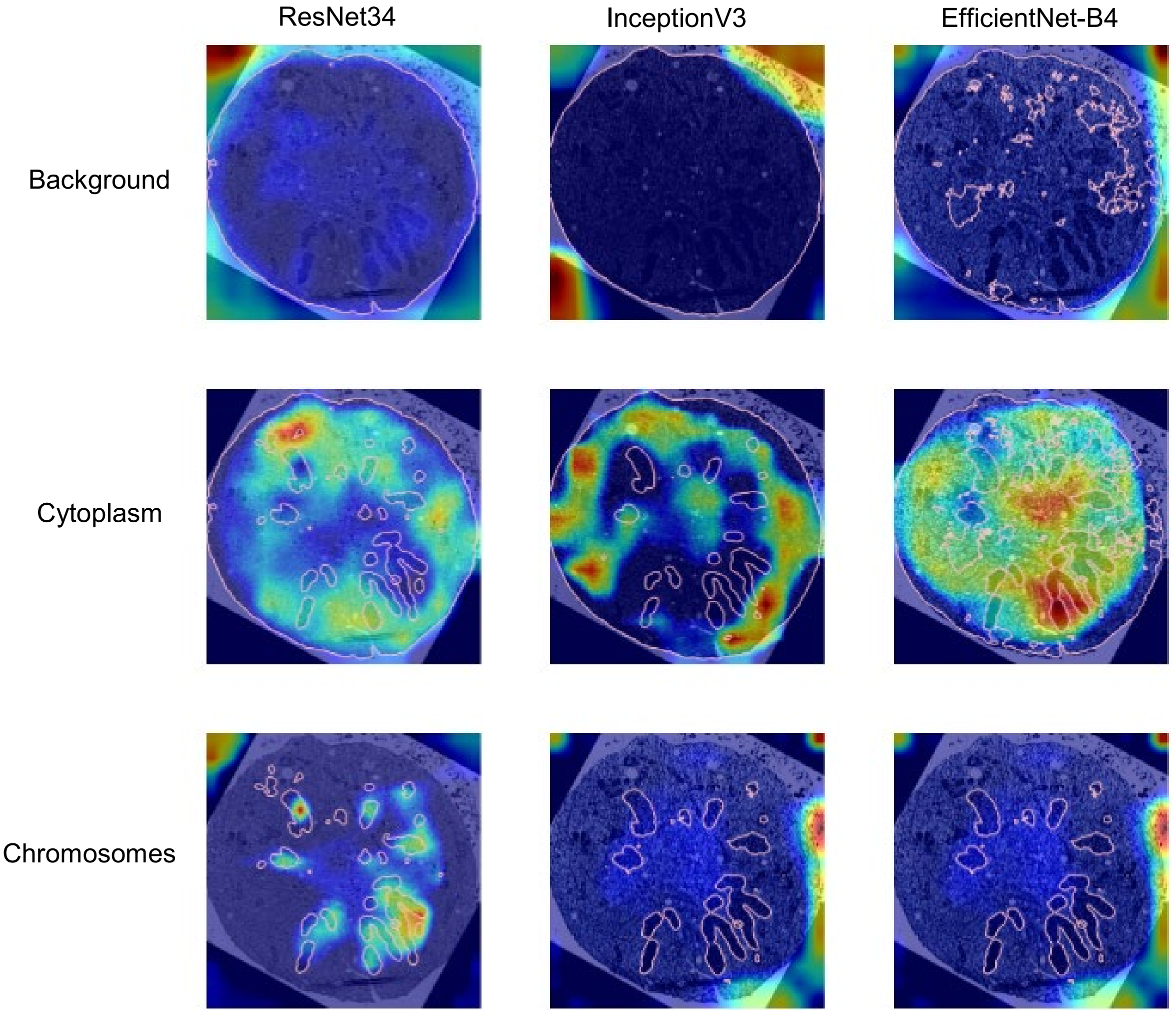

Representative Grad-CAM images from Dataset 3

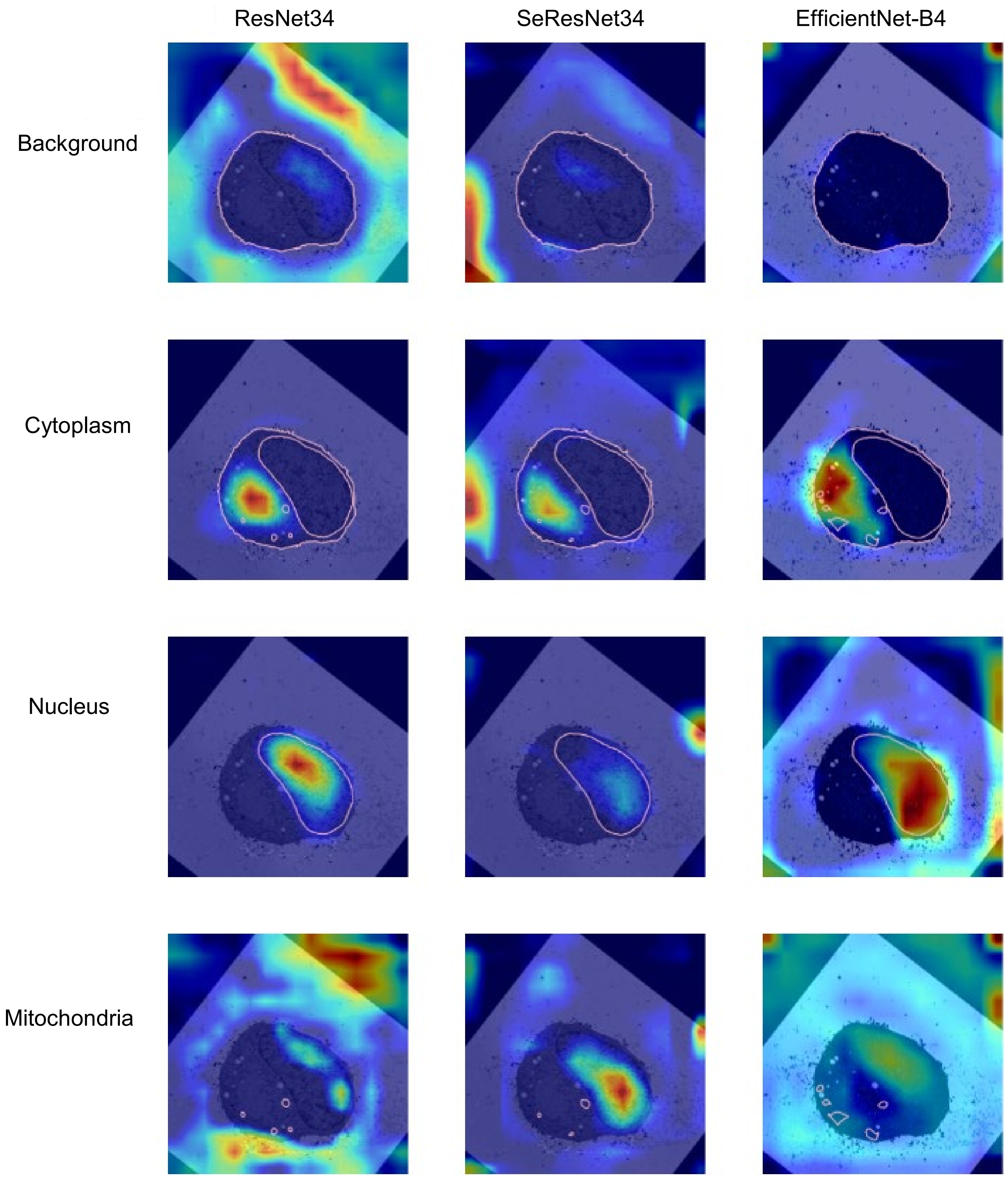

Representative Grad-CAM images from Dataset 4

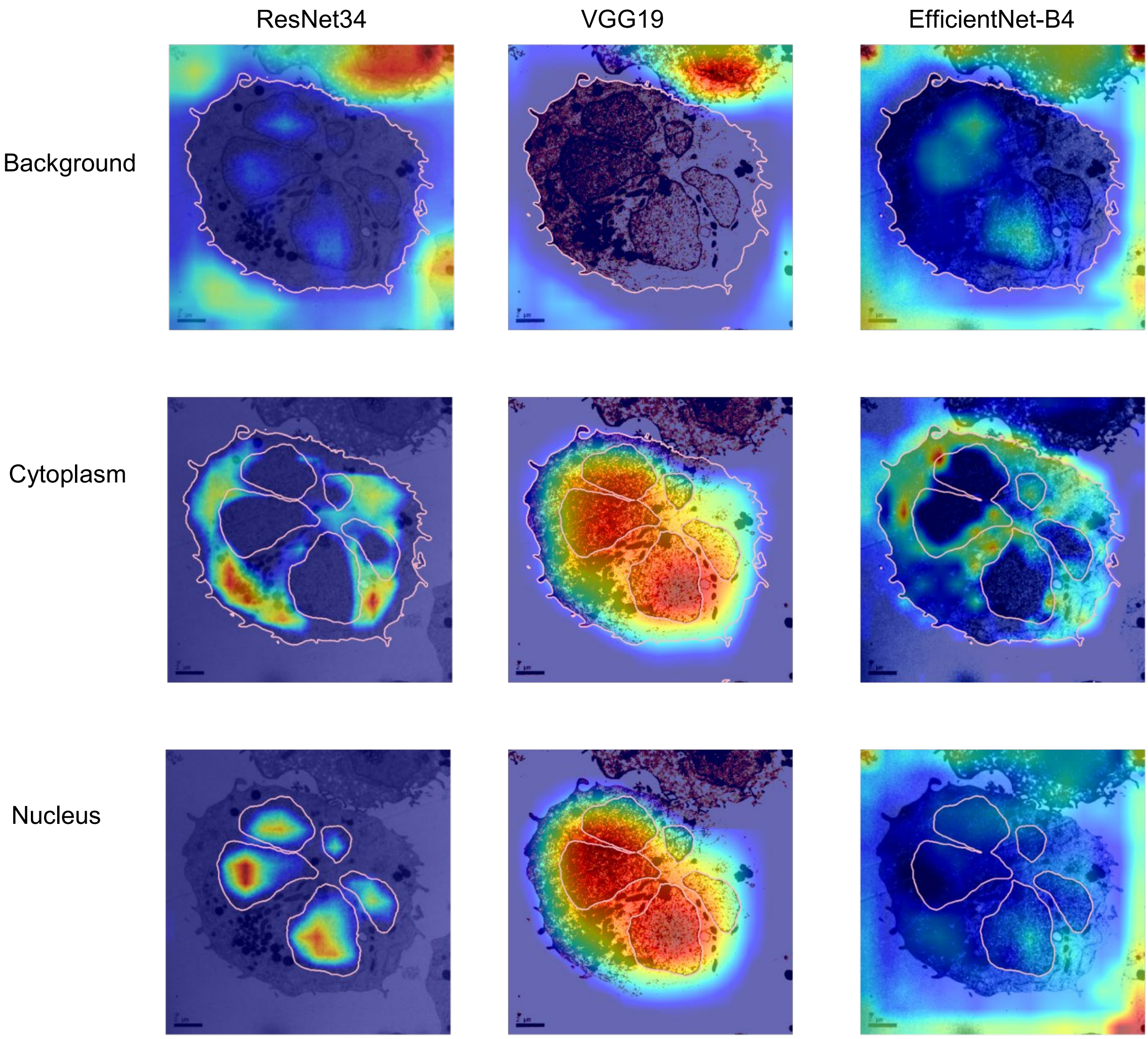

Representative Grad-CAM images from Dataset 5

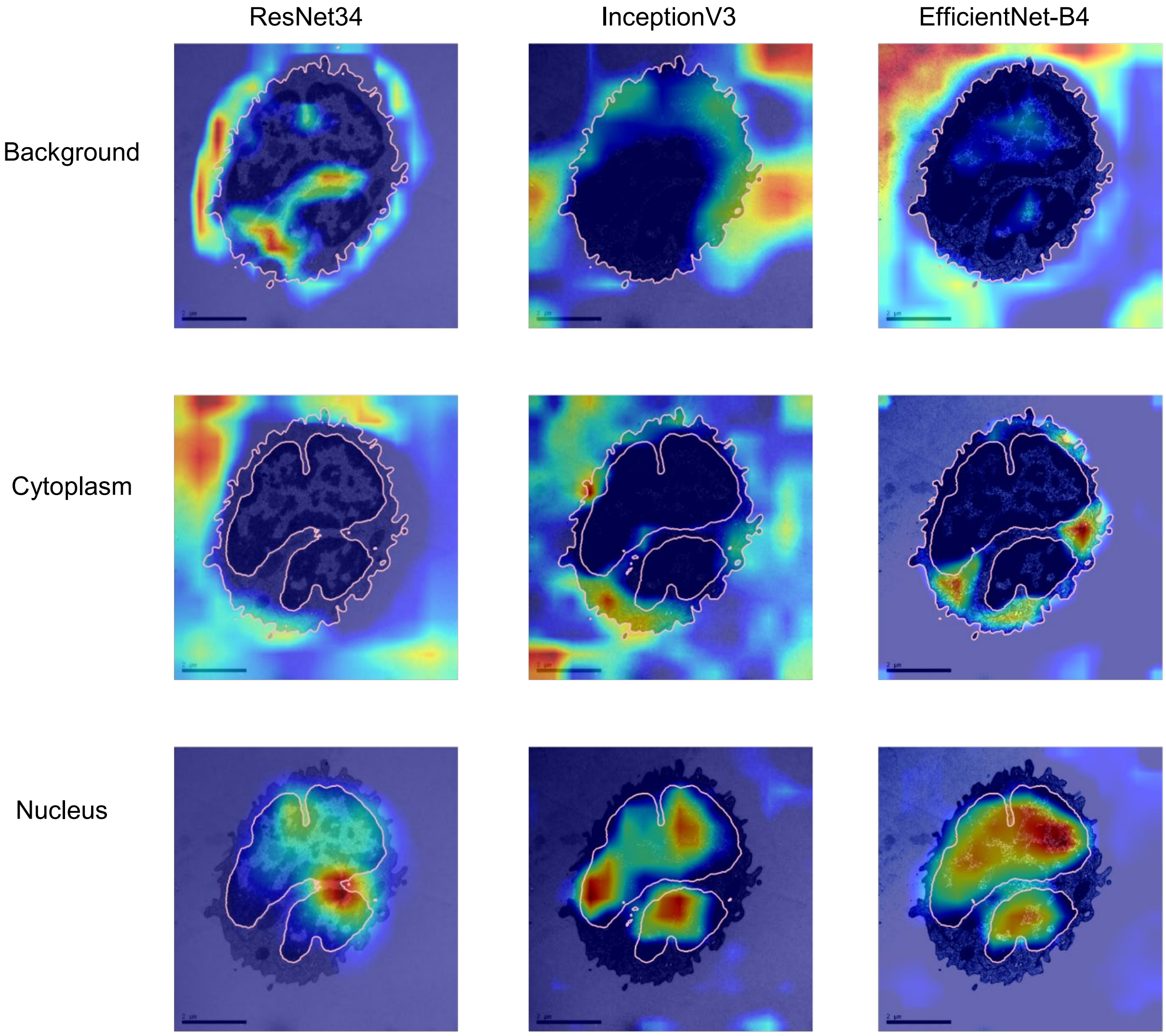

Representative Grad-CAM images from Dataset 6

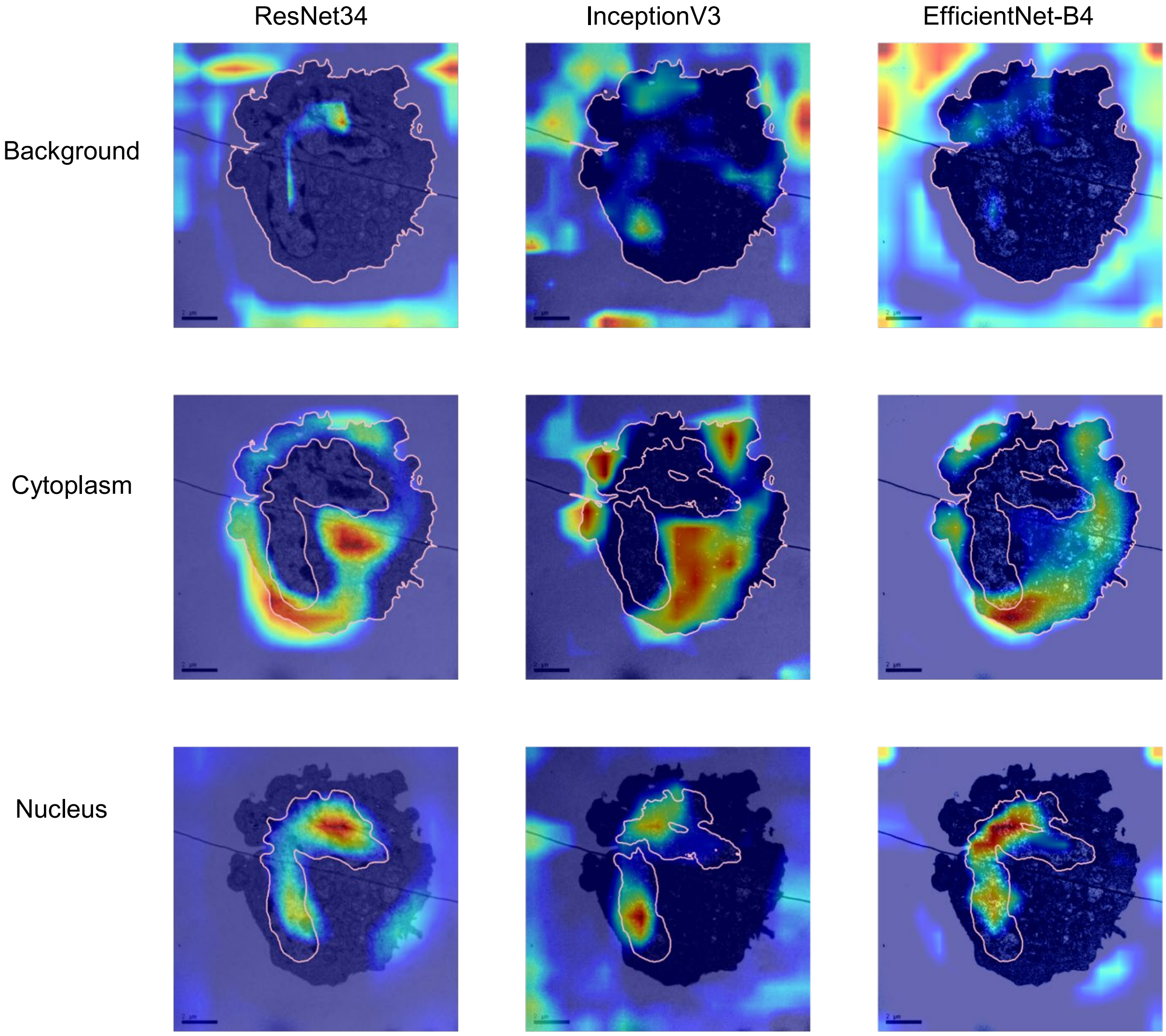

Representative Grad-CAM images from Dataset 7

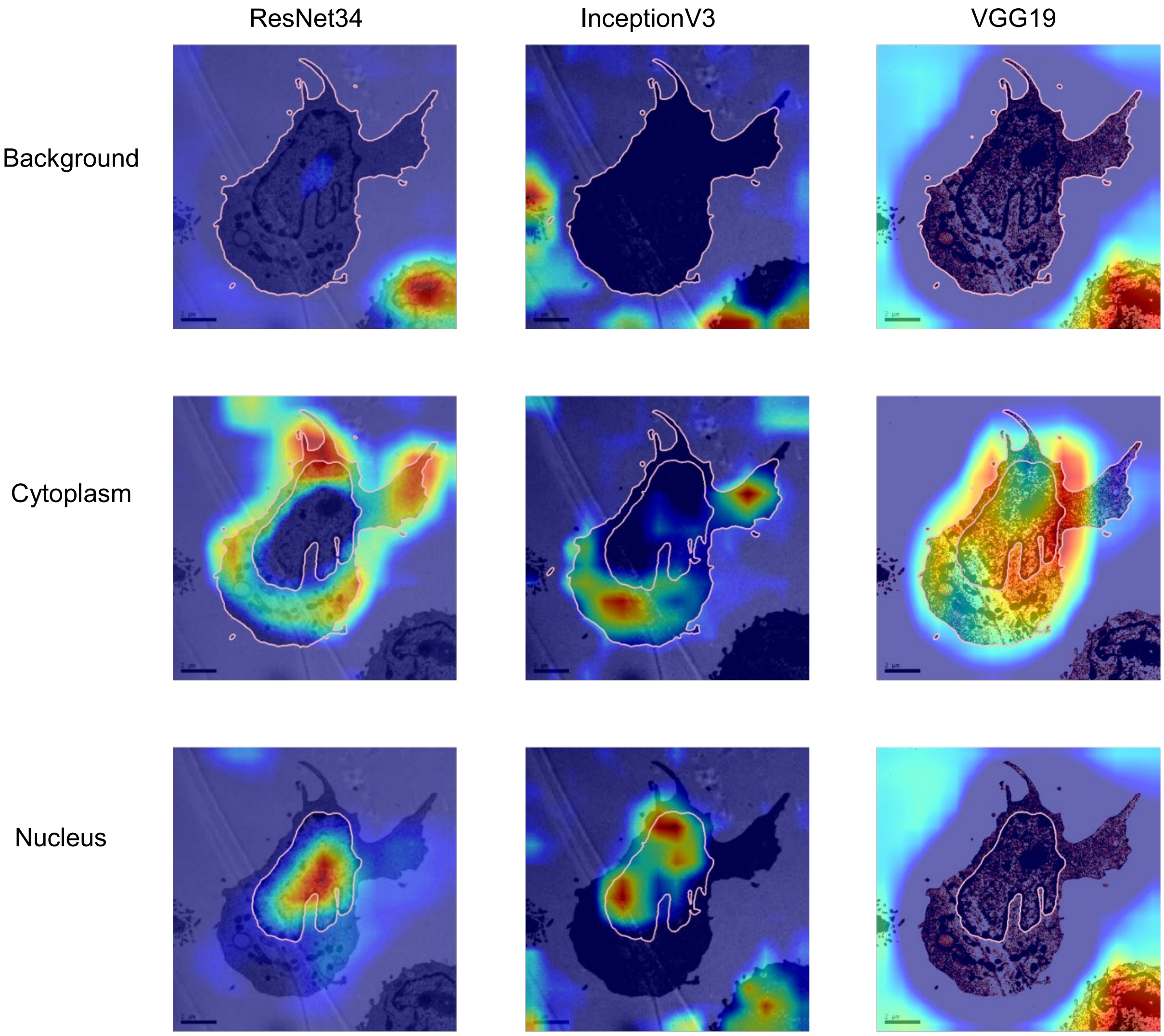

Supplement: Supplementary file 23 — Supplementary file23 (PDF 1198 KB) [file 418_2022_2148_MOESM23_ESM.pdf]
